# Pharmacologic degradation of WDR5 suppresses oncogenic activities of SS18::SSX and provides a therapeutic of synovial sarcoma

**DOI:** 10.1126/sciadv.ads7876

**Published:** 2025-04-23

**Authors:** Yao Yu, Xufen Yu, Bo Pan, Ho Man Chan, H. Ümit Kaniskan, Jian Jin, Ling Cai, Gang Greg Wang

**Affiliations:** ^1^ Department of Pharmacology and Cancer Biology, Duke University School of Medicine, Durham, NC 27710, USA.; ^2^ Departments of Pharmacological Sciences and Oncological Sciences, Tisch Cancer Institute, Icahn School of Medicine at Mount Sinai, New York, NY 10029, USA.; ^3^ Mount Sinai Center for Therapeutics Discovery, Icahn School of Medicine at Mount Sinai, New York, NY 10029, USA.; ^4^ Research and Early Development, Oncology R&D, AstraZeneca, Waltham, MA 02451, USA.; ^5^ Department of Pathology, Duke University School of Medicine, Durham, NC 27710, USA.; ^6^ Duke Cancer Institute, Duke University School of Medicine, Durham, NC 27710, USA.

## Abstract

Cancer-causing aberrations recurrently target the chromatic-regulatory factors, leading to epigenetic dysregulation. Almost all patients with synovial sarcoma (SS) carry a characteristic gene fusion, SS18::SSX, which produces a disease-specific oncoprotein that is incorporated into the switch/sucrose non-fermentable (SWI/SNF) chromatin-remodeling complexes and profoundly alters their functionalities. Targeting epigenetic dependency in cancers holds promise for improving current treatment. Leveraging on cancer cell dependency dataset, pharmacological tools, and genomic profiling, we find WDR5, a factor critical for depositing histone H3 lysine 4 (H3K4) methylation, to be an unexplored vulnerability in SS. Mechanistically, WDR5 and SS18::SSX interact and colocalize at oncogenes where WDR5 promotes H3K4 methylation and the chromatin association of SS18::SSX-containing chromatin-remodeling complexes. WDR5 degradation by proteolysis-targeting chimera (PROTAC) not only suppresses the SS18::SSX-related oncogenic programs but additionally causes the ribosomal protein deregulations leading to p53 activation. WDR5-targeted PROTAC suppresses SS growth in vitro and in vivo, providing a promising strategy for the SS treatment.

## Teaser

WDR5 as an SS vulnerability and WDR5 degradation by PROTAC are ways to suppress oncogenicity by SS18::SSX.

## INTRODUCTION

Rare and childhood cancers are often characterized by disease-specific gene fusions (i.e., onco-fusions) that recurrently cause chromatin and transcriptomic perturbations (*1*–*5*). For example, synovial sarcoma (SS) harbors a hallmark onco-fusion termed SS18::SSX (*6*, *7*), while the infantile, pediatric, and childhood leukemias frequently carry the onco-fusion involving lysine methyltransferase 2A [KMT2A, also known as mixed lineage leukemia 1 (MLL1)] (*2*, *8*) and nucleoporin 98 (*3*). Despite the fact that the targeted therapy drugs have revolutionized cancer treatment in general, with some becoming the dominant therapeutic modalities of certain common cancer types (*9*, *10*), little progress was made in developing targeted therapeutics for the rare yet aggressive cancers (*1*, *11*–*13*). In mice, SS18-SSX is sufficient in driving SS formation (*14*, *15*). Therefore, development of the therapeutic means to block onco-fusion–related tumorigenic functions holds a great promise for improving the current treatment of the affected patients.

Being one of the most common non-rhabdomyosarcomatous subtypes of soft tissue sarcoma (STS), SS accounts for approximately 5 to 10% of all STS cases (*1*, *11*–*13*). Typically, SS occurs first in the lower extremities near a joint, and as the disease progresses, it tends to infiltrate the nearby tissues such as the muscle and bones and then metastasizes to distant sites including the lung and lymph nodes (*16*, *17*). Currently, the mainstream treatments of SS include nonspecific radio- or chemotherapies and surgeries such as a limb-sparing procedure and limb amputation. Because of a lack of effective therapeutics and a high rate of metastasis, SS generally displays poor prognosis, with the 5-year survival rate of approximately 40 to 50% (*11*–*13*, *16*, *17*). Additional strategies are urgently needed to improve the treatment of SS. Almost all patients with SS harbor an aberrant chromosomal translocation that fuses the two genes, namely, SS translocation chromosome 18 (SS18, also known as SMARCL1, SYT, and SSXT) located in the chromosome 18q11 and one of the SSX family genes in the chromosome Xp11 (either SSX1, SSX2, or SSX4), producing a class of SS-specific onco-fusions termed SS18::SSX (*1*, *6*, *7*). In normal cells, SS18 functions as a subunit of switch/sucrose non-fermentable (SWI/SNF) chromatin-remodeling complexes; in SS cells, the SS18::SSX fusion protein is still able to be incorporated into SWI/SNF complexes but evicts the native SWI/SNF subunits, such as SS18 and the SWI/SNF related BAF chromatin remodeling complex subunit B1 (SMARCB1, also known as INI1 and BAF47), resulting in malfunction of various SWI/SNF remodeler complexes (*18*–*23*). For example, SS18::SSX was reported to aberrantly target the SWI/SNF remodeler complexes to developmental genes that are normally repressed by Polycomb repressive complex 1 (PRC1) and/or PRC2, an event that leads to abnormal gene activation (*18*, *19*, *21*, *22*). In agreement, SS was suggested to be a disease arising from the defects in cell lineage differentiation and/or acquisition of stemness (*18*, *19*, *24*). Overall, SS18::SSX profoundly deregulates the chromatin states and transcriptomic programs, driving the SS pathogenesis. Identification and targeting of epigenetic dependencies in SS shall provide a way to develop the mechanism-based therapies.

Leveraging on the cancer cell line dependency dataset, the medicinal chemistry tool compounds and integrated genomics and molecular oncology approaches, we here report the tryptophan-aspartic acid (W-D) repeat containing protein 5 (WDR5) to be a vulnerability and drug target in SS. WDR5, an integral component of the KMT2/MLL lysine methyltransferase complexes, is critical for the deposition of histone H3 lysine 4 mono-, di- and tri-methylation (H3K4me1/2/3) at cis-regulatory elements such as gene enhancers and promoters (*2*, *25*). We also unveil a previously unexplored interaction between WDR5 and SS18::SSX—The two physically associate with one another in the nucleoplasmic condensates and also exhibit a notable genome-wide colocalization at their target sites where the WDR5-containing and SS18::SSX-containing protein complexes act in concert to promote one another’s chromatin association and to activate the downstream oncogenic gene-expression programs. Using proteolysis-targeting chimera (PROTAC) technology, we have generated PROTACs for pharmacologically targeting and degrading WDR5 (*26*–*28*). Treatment of human SS cells with our lead WDR5 PROTAC degraders, MS67 and MS40 (*26*–*28*), potently degraded cellular WDR5 in SS and efficiently inhibited malignant growth of SS cells in vitro. Such SS-killing effects were not seen with the matched WDR5 protein-protein interaction (PPI) inhibitor or the designed PROTAC-inactive analog compounds, pointing to WDR5 degradation to be necessary for efficiently suppressing SS cell growth. In addition, the potent tumor-killing effect of MS67 was not seen in a panel of tested non-SS sarcoma cells and MS67 significantly suppressed the SS malignant growth in vivo using a cancer cell line–derived xenograft (CDX) model. Together, we report WDR5 to be an “epi” dependency in SS, a critical functional partner of SS18::SSX, and a highly valuable therapeutic target for improving the current treatment of patients with SS.

## RESULTS

### WDR5 is an epigenetic dependency in SS

**Fig. 1.**
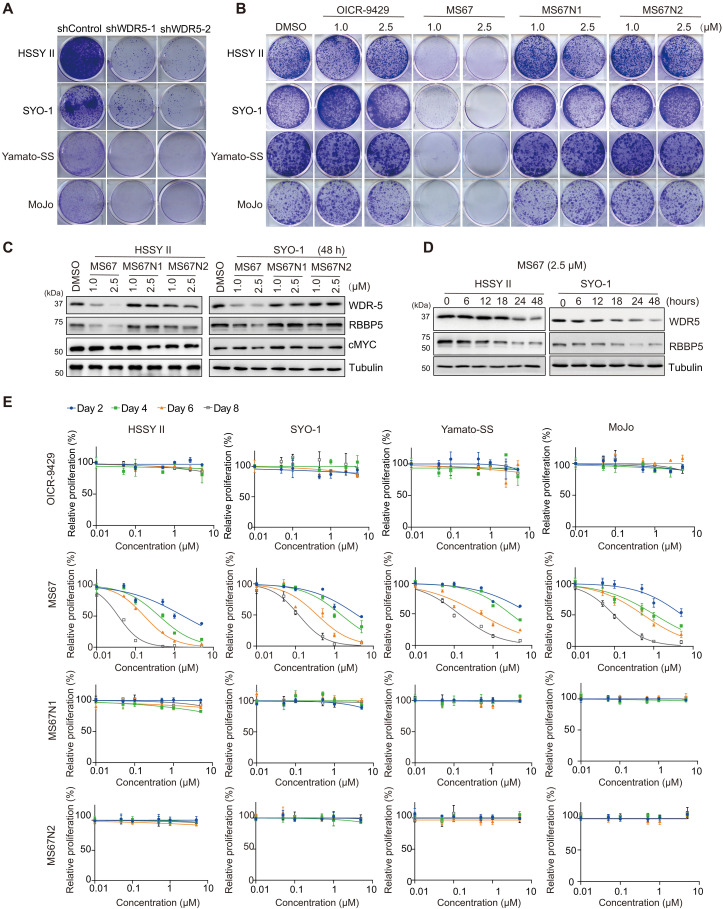
MS67 potently and selectively inhibits malignant growth of SS cells. (**A** and **B**) Images of colony formation using the indicated human SS cells (HSSY II, SYO-1, Yamato-SS, and MoJo), stably transduced with either a control shRNA or the ones targeting WDR5 (shWDR5-1 or shWDR5-2) (A), or the parental SS cells grown in the presence of the indicated concentration of DMSO, OICR-9429, MS67, MS67N1, or MS67N2 (B). All experiments were repeated at least twice, with representative results shown here. (**C** and **D**) WB of WDR5, RBPP5, c-MYC, and tubulin in HSSY II (left) and SYO-1 cells (right) treated with the indicated concentration of DMSO, MS67, MS67N1, or MS67N2 for 48 hours (h) (C), or with 2.5 μM of MS67 for the indicated duration (D). All experiments were repeated at least twice, with representative results shown here. (**E**) Plots of growth inhibition using the indicated SS cells, treated with a range of concentration (*x* axis) of either OICR-9429 (top), MS67 (second row), MS67N1 (third row), or MS67N2 (bottom) for 2, 4, 6, or 8 days. *Y* axis, presented in the means ± SEM of replicated data, shows the relative growth after normalization of the cell number to the DMSO-treated controls (*n* = 3 independent experiments).

SS18::SSX perturbs appropriate chromatin regulation, leading to SS pathogenesis. To uncover epigenetic dependencies in SS, we used the publicly available datasets and plotted the dependency scores for all epigenetic factors in the commonly used SS cell lines (fig. S1, A and B; also see Materials and Methods). When compared with non-SS sarcoma cell lines, the three SS lines (HSSY II, an SS18::SSX1-positive SS cell line, as well as SYO-1 and FUJI, the two SS18::SSX2-positive SS lines) exhibited notable dependencies on both bromodomain-containing 9 (BRD9) (fig. S1A, *y* axis), an SWI/SNF chromatin-remodeling complex component previously reported to be an SS dependency (*29*), and WDR5 (fig. S1A, *x* axis), an integral component of the H3K4 methylation-depositing KMT2/MLL complexes (*2*, *25*). For example, WDR5 is the epi factor showing the highest dependency score in SYO-1 cells (fig. S1B). Yet, a potential SS-promoting role for WDR5 has not been carefully studied to date. To testify the involvement of WDR5 for SS growth, we introduced either one of the two WDR5-specific short hairpin RNAs (shRNAs) to a panel of human SS cell lines carrying SS18::SSX (HSSY-II, SYO-1, Yamato-SS, and MoJo) to induce knockdown (KD) of WDR5 (fig. S1C), and in all cases, the WDR5 shRNA dramatically inhibited colony formation when compared with controls (Fig. 1A). Furthermore, we used a set of WDR5-targeting small molecules (fig. S1D), including OICR-9429 (a small-molecule inhibitor of WDR5 that competitively blocks the PPI between WDR5 and partner such as KMT2A/MLL1) (*26*), MS67 (an OICR-9429– and VHL-based WDR5 PROTAC degrader) (*26*), MS67N1 (a diastereoisomer and inactive control of MS67, which contains the identical WDR5-binding moiety and linker but shows the abrogated binding to VHL, aka, MS67N) (*26*), and MS67N2 (another inactive analog of MS67 that shows the decreased binding to WDR5 but intact binding to VHL that we developed in this work). Here, only the treatment with MS67, but not OICR-9429 or the two PROTAC-inactive analogs of MS67 (MS67N1 and MS67N2), decreased the colony formation of tested SS cells (Fig. 1B). We also measured the dose-dependent and time-dependent effects of used compounds. First, Western blots (WB) showed that MS67 induced the degradation of WDR5 and retinoblastoma (RB)-binding protein 5 (RBBP5), a WDR5-associated KMT2/MLL complex component, in a concentration-dependent and time-dependent manner in tested SS cells, the effects not seen with MS67N1 or MS67N2 (Fig. 1, C and D). cMyc, another oncoprotein reported to be associated with WDR5 in cancer (*30*), was not substantially affected by the MS67 treatment in SS cells (Fig. 1C, see cMyc). Consistent with what was seen in the colony-forming assays, the proliferation-based assessment showed that MS67 had potent, consistent antiproliferation effects in all tested SS cell lines, while OICR-9429, MS67N1, and MS67N2 generally showed no or very little effect on tumor cell growth (Fig. 1E). In addition, WDR5 degradation by MS67 had little or very mild effects on the growth of a panel of tested non-SS sarcoma lines, such as Ewing sarcoma (A673 and RD-ES cells), rhabdomyosarcoma (A204, Rh4, and Rh10 cells), and osteosarcoma (U2OS cells) (fig. S1E), despite the comparable WDR5 degradation by MS67 in these non-SS sarcoma lines when compared with SS cells (fig. S1F). The half maximal effective concentration (EC_50_) values of MS67 measured in SS cells were within a nanomolar-to-submicromolar range, whereas EC_50_ values of MS67N1 and MS67N2 in the same SS cells and those of MS67 in the tested non-SS sarcoma lines went beyond the assessed concentration range and could not be confidently determined (fig. S1G). In addition, we validated the effect of WDR5-targeting PROTAC by using MS40, an independent PROTAC designed on the basis of OICR-9429 and pomalidomide, a different E3 ligand recruiting cereblon (*27*)—When compared to dimethyl sulfoxide (DMSO), the treatment of MS40, but not its matched E3-inactive and WDR5-binder–inactive analogs (MS40N1 and MS40N2) (*27*), effectively degraded WDR5 in HSSY II cells (fig. S1H), which also dramatically decreased malignant growth of SS cells in both the colony formation–based (fig. S1I) and proliferation-based assays (fig. S1J). Together, our results demonstrated an unexplored WDR5 dependency in SS.

### WDR5 interacts with and colocalizes with the SS18::SSX-harboring SWI/SNF complexes in SS

**Fig. 2.**
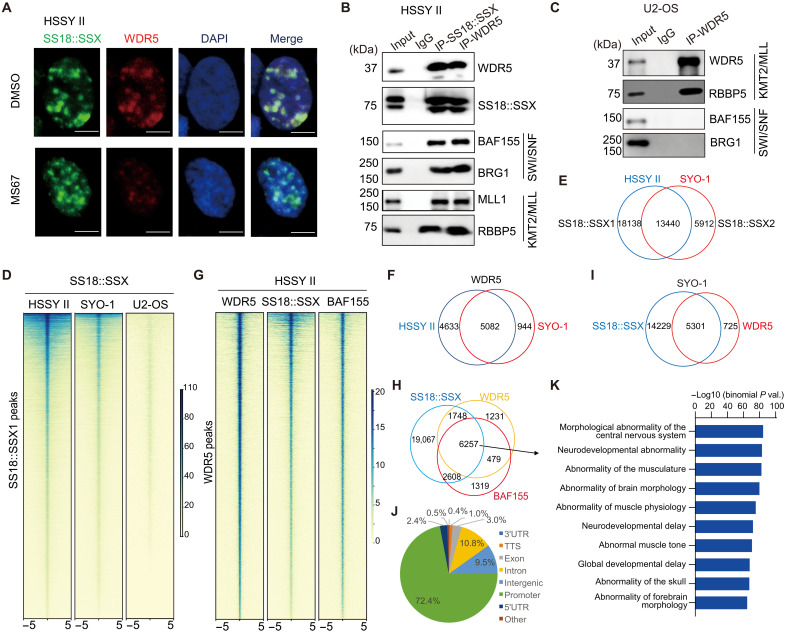
WDR5 colocalizes with SS18::SSX genome-wide in SS cells. (**A**) Representative co-IF images of SS18::SSX, WDR5 and DNA [probed with 4′,6-diamidino-2-phenylindole (DAPI)] in HSSY II cells, treated with 2.5 μM of DMSO (top) or MS67 (bottom) for 48 hours. Scale bar, 5 μm. The experiment was repeated at least twice, with representative results shown here. (**B** and **C**) co-IP to detect interaction between WDR and the SWI/SNF complex components in HSSY II (B) or U2OS cells (C) using either nonspecific IgG (lane 2), anti-SS18::SSX [lane 3 in (B)], or anti-WDR5 antibody [lane 4 in (B) and lane 3 in (C)]. Input was loaded to lane 1 as loading control. The experiment was repeated at least twice, with representative results shown here. (**D**) Heatmaps showing the SS18::SSX CUT&Tag signal densities in either HSSY II, SYO-1, or U2OS cells, ±5 kb from the centers of SS18::SSX peaks in HSSY II cells. (**E** and **F**) Venn diagram using the SS18::SSX peaks (E) or WDR5 peaks (F) called in HSSY II and SYO-1 cells. (**G**) Heatmaps showing the WDR5, SS18::SSX, and SMARCC1/BAF155 CUT&Tag signal intensities, ±5 kb from the centers of WDR5 peaks, in HSSY II cells. (**H** and **I**) Venn diagram showing overlap of SS18::SSX with the WDR5 and/or SMARCC1/BAF155 peaks in HSSY II (H) or SYO-1 (I) cells. (**J** and **K**) Genomic annotation (J) and gene ontology (GO) analysis (K) using the peaks cobound by SS18::SSX, WDR5, and SMARCC1/BAF155 in HSSY II cells as defined in (H). *Y* axis in (K) shows the −log_10_ value of binomial *P* values.

Having known that WDR5 is critically involved in malignant growth of SS cells, we next aimed to dissect its function in this disease. First, immunofluorescence (IF) of SS18::SSX in HSSY II cells readily detected a pattern of condensates or puncta in the nucleoplasm (fig. S2A, top), consistent to previous reports that SS18::SSX can phase separate, either by itself or with associated partners (*31*, *32*). As a negative control, IF with the same anti-SS18::SSX antibody in U2OS cells, an osteosarcoma line lacking SS18::SSX expression, detected little signals (fig. S2B). In addition, co-IF detected that WDR5 not only exhibits a similar condensation pattern but also demonstrates notable colocalization with SS18::SSX in HSSY II cells (fig. S2A and Fig. 2A, top, in two separate experiments). While the treatment with MS67 readily abolished the WDR5 foci, it kept those condensates of SS18::SSX largely intact (Fig. 2A, bottom)—Neither the total number nor the averaged size of the SS18::SSX puncta was significantly affected by MS67 versus mock treatment (fig. S2, C and D). These results suggested an unexplored interaction between SS18::SSX and WDR5 in SS cells. To test this idea, we then conducted coimmunoprecipitation (co-IP) in HSSY II cells—Following the pulldown of SS18::SSX but not the mock IP, we detected not only the SWI/SNF complex components, such as the SWI/SNF-related BAF chromatin remodeling complex subunit C1 (SMARCC1/BAF155) and SWI/SNF-related BAF chromatin remodeling complex subunit ATPase 4 (SMARCA4/BRG1) but also WDR5 and the WDR5-associated KMT2/MLL complex subunits (such as KMT2A/MLL1 and RBBP5) (Fig. 2B, lanes 3 versus 2). Conversely, IP using the anti-WDR5 versus nonspecific immunoglobulin G (IgG) antibody also detected strong interaction of WDR5 with not only the KMT2/MLL complex components but also SS18::SSX and associated SWI/SNF subunits (Fig. 2B, lane 4 versus 2). Co-IP using the same anti-WDR5 antibody in U2OS cells readily pulled down RBBP5 but not SMARCC1/BAF155 or SMARCA4/BRG1 (Fig. 2C). Thus, there exists an onco-fusion (SS18::SSX)–dependent association between the KMT2/MLL and SWI/SNF complexes in SS.

Having demonstrated the physical interaction between WDR5 and the SS18::SSX1-containing SWI/SNF complexes, we next assessed whether the two colocalize at a genome-wide scale. First, we performed Cleavage Under Targets and Tagmentation (CUT&Tag) (*33*) for SS18::SSX. To ascertain suitability and specificity of the used anti-SS18::SSX antibody, we applied it for CUT&Tag in three cell lines—the SS18::SSX1-positive HSSY II cells, the SS18::SSX2-positive SYO-1 cells, and the SS18::SSX-negative U2OS cells. As expected, we observed the expected low and negligible signals in U2OS cells (Fig. 2D, the third versus the first two columns), as exemplified by what was observed at key SS oncogenes (*18*, *19*, *29*), *motor neuron and pancreas homeobox 1* (*MNX1*) and *SRY-box transcription factor 8* (*SOX8*) (fig. S2E). In contrast, there were robust and consistent CUT&Tag peaks of SS18::SSX in both HSSY II and SYO-1 cells (Fig. 2, D and E). Such a specificity of the anti-SS18::SSX antibody was consistent with a lack of IF signals in U2OS cells (fig. S2B) and a prior report assessing clinical SS samples with this antibody (*34*). In addition, we conducted Cleavage Under Targets and Release Using Nuclease (CUT&RUN) (*35*) for SS18::SSX and WDR5. Here, we found that the SS18::SSX and WDR5 signals detected by CUT&Tag and CUT&RUN in the same HSSY II cells are highly correlated (fig. S2F), with the called SS18::SSX and WDR5 peaks showing significant overlap (fig. S2G), which confirmed validity of our genomic mapping strategies. Next, we conducted CUT&Tag for WDR5 and/or SMARCC1/BAF155 in HSSY II and SYO-1 cells (fig. S2H) and found that the WDR5 peaks identified in the two independent SS cells overlap significantly (Fig. 2F); furthermore, a vast majority of WDR5 peaks overlapped SS18::SSX and/or BAF155 in HSSY II (Fig. 2, G and H) and SYO-1 cells (Fig. 2I). The peaks cobound by WDR5 and the SS18::SSX-containing SWI/SNF complexes were mainly found at gene promoters (Fig. 2J) and displayed the marked enrichment for developmental genes such as those related to the development of the nervous system, muscle, and brain (Fig. 2K). Unbiased motif search analysis using these WDR5- and SS18::SSX-cobound peaks uncovered a strong enrichment for the consensus binding sites of transcription factors (TFs) known to be essential for embryonic development and neurogenesis, such as the NFY, SP/KLF, and E-box families of TFs including cMyc (fig. S2I). Collectively, our integrated co-IF, co-IP, and genome-wide profiling lend a strong support for the physical association between the WDR5-harboring KMT2A/MLL complexes and SS18::SSX-associated SWI/SNF complexes, suggesting an unexplored functional cross-talk between the two during the SS oncogenesis.

### WDR5 is required for chromatin binding by SS18::SSX and SS18::SSX-associated SWI/SNF complexes in SS

**Fig. 3.**
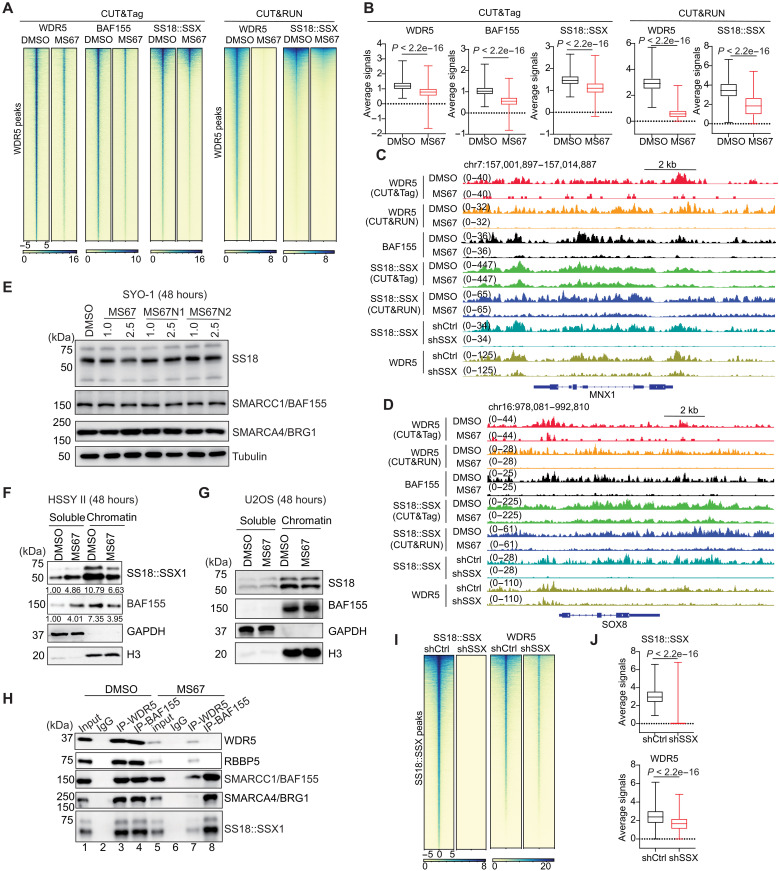
WDR5-targeting PROTAC decreases the chromatin binding by SS18::SSX and the SWI/SNF chromatin-remodeling complex in SS. (**A** and **B**) Heatmaps (A) and box plot of averaged intensities (B) of the indicated CUT&Tag or CUT&RUN signals (after normalization to spike-in control) of WDR5, SS18::SSX, or SMARCC1/BAF155 (±5 kb from the peak centers) in HSSY II cells, treated with 2.5 μM of DMSO or MS67 for 4 days. Wilcoxon test was used to generate *P* value. (**C** and **D**) Integrative Genomics Viewer (IGV) views of the indicated CUT&Tag or CUT&RUN signals at *MNX1* (C) and *SOX8* (D) in HSSY II cells, treated as in (A), or transduced with a control (shCtrl) or SS18::SSX-targeting shRNA (shSSX). (**E**) WB of SWI/SNF complex components using SYO-1 cells, treated with the indicated concentration of drugs for 48 hours. (**F** and **G**) WB of the indicated protein in the soluble nucleoplasmic (left) or chromatin-bound (right) fraction of HSSY II (F) or U2OS (G) cells, treated with 2.5 μM of DMSO or MS67 for 48 hours. Quantification of protein levels in (F) was normalized to soluble-DMSO fraction samples and labeled under the gel image. (**H**) Co-IP for interaction between WDR5 and the SS18::SSX-containing SWI/SNF complexes in HSSY II cells, first treated with 2.5 μM of DMSO (lanes 1 to 4) or MS67 (lanes 5 to 8) for 48 hours and then subject to IP using either nonspecific IgG, anti-WDR5, or anti-SS18::SSX antibody. WB and co-IP were repeated at least twice, with representative results shown here. (**I** and **J**) Heatmaps (I) and averaged intensities (J) of CUT&Tag signals (normalized to spike-in controls) of SS18::SSX and WDR5 (±5 kb from the peak centers) in HSSY II cells, transduced with the control (shCtrl) or SSX-targeting shRNA (shSSX). The *y* axis in (J) represents the average CUT&Tag signals and Wilcox test was used to generate *P* value.

Having observed a notable genome-wide colocalization between WDR5 and SS18::SSX, we next aimed to assess whether WDR5 is essential for functionalities of SS18::SSX and the associated SWI/SNF complexes in SS cells. Toward this end, we treated the HSSY II cells with either DMSO or MS67 and conducted CUT&Tag or CUT&RUN for SS18::SSX, WDR5 and SMARCC1/BAF155. Here, CUT&Tag and CUT&RUN were conducted with the addition of spike-in controls, allowing for a quantitative comparison of signals across samples (for details, see Materials and Methods). As expected, the treatment with MS67 decreased the overall chromatin binding of WDR5 when compared to mock (fig. S3A and Fig. 3, A and B, see panels of WDR5). Concurrently, we detected the significant decreases in overall binding of both SS18::SSX and SMARCC1/BAF155 at the same genomic target sites upon the treatment of MS67 versus mock, regardless of CUT&Tag or CUT&RUN being used (fig. S3A and Fig. 3, A and B; see panels of SS18::SSX and BAF155). The MS67-elicited effects on diminishing the binding of WDR5, SS18::SSX and BAF155 were clearly observed at a suite of the previously reported SS oncogenes and/or SS18::SSX targets (*18*, *19*, *29*, *36*), such as *MNX1*, *SOX8*, *neurotensin receptor 1* (*NTSR1*), and *SIM bHLH transcription factor 2* (*SIM2*) (Fig. 3, C and D, and fig. S3B). In addition, we conducted WB after the treatment of DMSO, MS67, or the matched PROTAC-inactive analogs (MS67N1 and MS67N2) and found that these treatments did not affect global levels of SS18::SSX and the tested SWI/SNF complex subunits, SMARCC1/BAF155 and SMARCA4/BRG1, in both HSSY II (fig. S3C) and SYO-1 cells (Fig. 3E). Next, we performed the chromatin fractionation assays in the HSSY II cells and found that, compared with DMSO, the treatment of MS67 led to a decrease in the chromatin binding by SMARCC1/BAF155 and SMARCA4/BRG1 (Fig. 3F, right), as well as the concurrent increase of the two in the soluble nucleoplasmic fraction (Fig. 3F, left). The chromatin fractionation assay using U2OS cells, which do not express SS18::SSX, showed that the same treatment of MS67 did not have notable impact on the overall chromatin binding of normal SS18 and SMARCC1/BAF155 (Fig. 3G), consistent with a lack of interaction between WDR5 and the SWI/SNF complexes in this non-SS line (Fig. 2C).

Having observed the effect of WDR5 PROTAC on decreasing the chromatin binding by the SS18::SSX-containing SWI/SNF complexes, we further queried whether WDR5 PROTAC influences the integrity of this complex. Here, we treated the HSSY II cells with the same concentration of MS67 versus DMSO as the above experiment, followed by co-IP and WB. We confirmed an expected dramatic loss of both WDR5 and RBBP5 following the MS67 treatment versus mock (Fig. 3H, input lanes 5 versus 1). WB after anti-SMARCC1/BAF155 IP showed that the MS67-induced degradation of WDR5 did not influence the interaction of SMARCC1/BAF155 with SS18::SSX and SMARCA4/BRG1 (Fig. 3H, lanes 8 versus 4). As a procedure control, the anti-WDR5 IP in the same MS67-treated cells did not pull down much of WDR5 and RBBP5 because of degradation of the latter, and it also failed to efficiently pull down SS18::SSX or the tested SWI/SNF complex components (Fig. 3H, lanes 7 versus 3).

Moreover, we asked whether SS18::SSX conversely regulates the chromatin binding of WDR5. Toward this direction, we conducted the SS18::SSX KD in HSSY II cells using the previously validated SSX-targeting shRNAs (fig. S3D) (*19*), followed by CUT&Tag of SS18::SSX and WDR5. When compared to mock, the chromatin binding of SS18::SSX in the SS18::SSX KD cells exhibited an expected genome-wide loss (Fig. 3, I and J; see panels of SS18::SSX; after normalization to the spike-in control signals). Concurrently and upon SS18::SSX KD, the spike-in–controlled CUT&Tag of WDR5 demonstrated a global decrease in chromatin binding as well (Fig. 3, I and J; see panels of WDR5). Thus, SS18::SSX can promote the WDR5 binding onto the targeted chromatin sites.

Recently, it has been reported that the interaction of SSX, which is gained by the SS18::SSX fusion, with histone H2A lysine 119 mono-ubiquitination (H2AK119ub), a Polycomb-associated repressive histone mark, serves as one of the primary mechanisms for retargeting of the SS18::SSX-harboring remodeler complexes to Polycomb-targeted genomic sites (*21*, *22*). To illustrate whether the WDR5 PROTAC-caused decrease in the chromatin binding of SS18::SSX is due to the H2AK119ub dysregulation, we performed the H2AK119ub CUT&Tag. Here, there were no notable substantial changes of H2AK119ub at the SS18::SSX-binding peaks after the treatment of HSSY II cells with MS67 when compared to DMSO or PROTAC-inactive analogs (fig. S4).

Together, there exists a functional interaction between SS18::SSX and WDR5 in SS, which potentiates optimal chromatin occupancies of their associated complexes. Such an interaction between the SWI/SNF and WDR5-containing complexes is lacking in the SS18::SSX-negative U2OS cells and thus appears to rely on the presence of SS18::SSX, which agrees with the quite selective killing effects of WDR5-degrading PROTAC on SS cells, when compared to non-SS sarcoma cells (fig. S1).

### WDR5 is critical for modulating the appropriate chromatin state at SS18::SSX’s target sites in SS

**Fig. 4.**
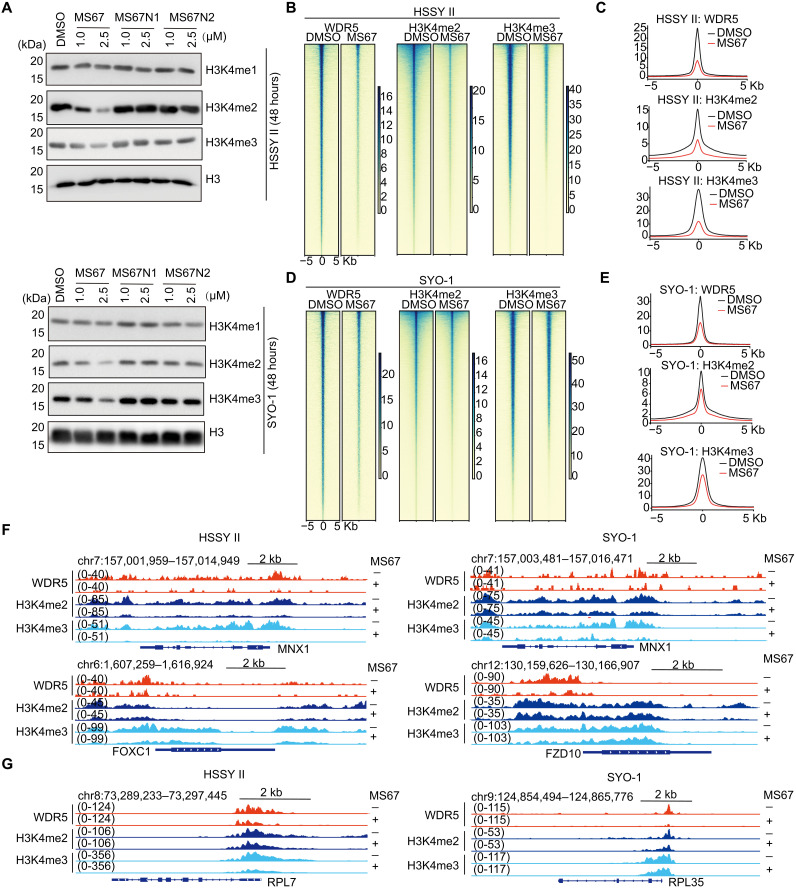
WDR5 PROTAC suppresses H3K4me2/3 on the WDR5-targeted genomic sites in SS cells. (**A**) Immunoblotting of H3K4me1, H3K4me2, and H3K4me3 in HSSY II (top) and SYO-1 cells (bottom), treated with the indicated concentration of DMSO, MS67, MS67N1, or MS67N2 for 48 hours. WB experiments above were repeated at least twice, with representative results shown here. (**B** to **E**) Heatmaps [(B) and (D)] and averaged intensities [(C) and (E)] of WDR5, H3K4me2, and H3K4me3 CUT&Tag signals after normalization to the spike-in control signals, ±5 kb from the centers of the called peaks, in HSSY II [(B) and (C)] and SYO-1 [(D) and (E)] cells treated by 2.5 μM of DMSO or MS67 for 4 days. (**F** and **G**) IGV views of the indicated developmental and stemness genes (F), as well as RP genes (G), in the DMSO (MS67^−^)– or MS67 (MS67^+^)–treated HSSY II (left) and SYO-1 cells (right).

The KMT2/MLL-WDR5 family complexes are critically involved in deposition of H3K4 methylation at target gene promoters and enhancers, which also cross-talk with other chromatin modulators, such as PRC1, PRC2, and chromatin-remodeling complexes, to promote and sustain transcriptional competence, activation, and/or elongation (*25*, *37*, *38*). Consistent with such a notion, the treatment of MS67, and not MS67N1 and MS67N2, led to the global decreases of H3K4me2 and H3K4me3, and not H3K4me1, in HSSY II and SYO-1 cells (Fig. 4A). To systematically define the site-specific chromatin-modulating effect of WDR5 PROTAC in SS, we further performed CUT&Tag of H3K4me3 and H3K4me2 after the treatment of HSSY II and SYO-1 cells with MS67 versus DMSO. In the mock-treated cells, we found a vast majority of WDR5 peaks to be cobound by H3K4me3 and H3K4me2 both (fig. S5, A and B). In agreement with previous studies (*26*, *39*), the gene ontology (GO) analysis of genes associated with the WDR5- and H3K4me3/2-cobound peaks identified the signatures of ribosomal protein (RP)–coding genes and gene targets of the chromatin-modifying complexes (such as Polycomb and KMT2/MLL) among the most significant enriched categories (fig. S5C). Compared to mock, the MS67 treatment led to a significant decrease in the overall bindings of WDR5, H3K4me3, and H3K4me2 at their cotargeted sites in both HSSY II (Fig. 4, B and C, and fig. S5, D) and SYO-1 cells (Fig. 4, D and E). As a result, approximately 82 and 65% of all WDR5 peaks were removed in HSSY II and SYO-1 cells, respectively, after the MS67 treatment (fig. S5, E and F). Such an MS67-caused reduction of H3K4me3 and H3K4me2 was obvious at the SS oncogenes and developmental genes (*18*, *19*, *29*) such as *MNX1*, *forkhead box C1* (*FOXC1*), and *frizzled class receptor 10* (*FZD10*) (Fig. 4F), as well as RP genes such as ribosomal protein L7 (*RPL7*) and ribosomal protein L35 (*RPL35*) (Fig. 4G), which were known to be critical targets of WDR5 in cancers (*26*, *40*). We also integrated CUT&Tag datasets of SS18::SSX, SMARCC1/BAF155, WDR5, and H3K4me3/2. Here, both Venn diagram (fig. S5G) and Pearson correlation coefficient plots (fig. S5H) showed them to be positively correlated, with approximately 85% of SS18::SSX- and SMARCC1/BAF155-cobound peaks to be cooccupied by H3K4me2 and H3K4me3 as well (fig. S5G). Together, the WDR5-harboring KMT2/MLL complexes play a critical role in modulating the chromatin landscape at target sites of SS18::SSX in SS.

### WDR5 degradation in SS suppresses the overall transcription of SS oncogenes, developmental genes, and RP-coding genes

**Fig. 5.**
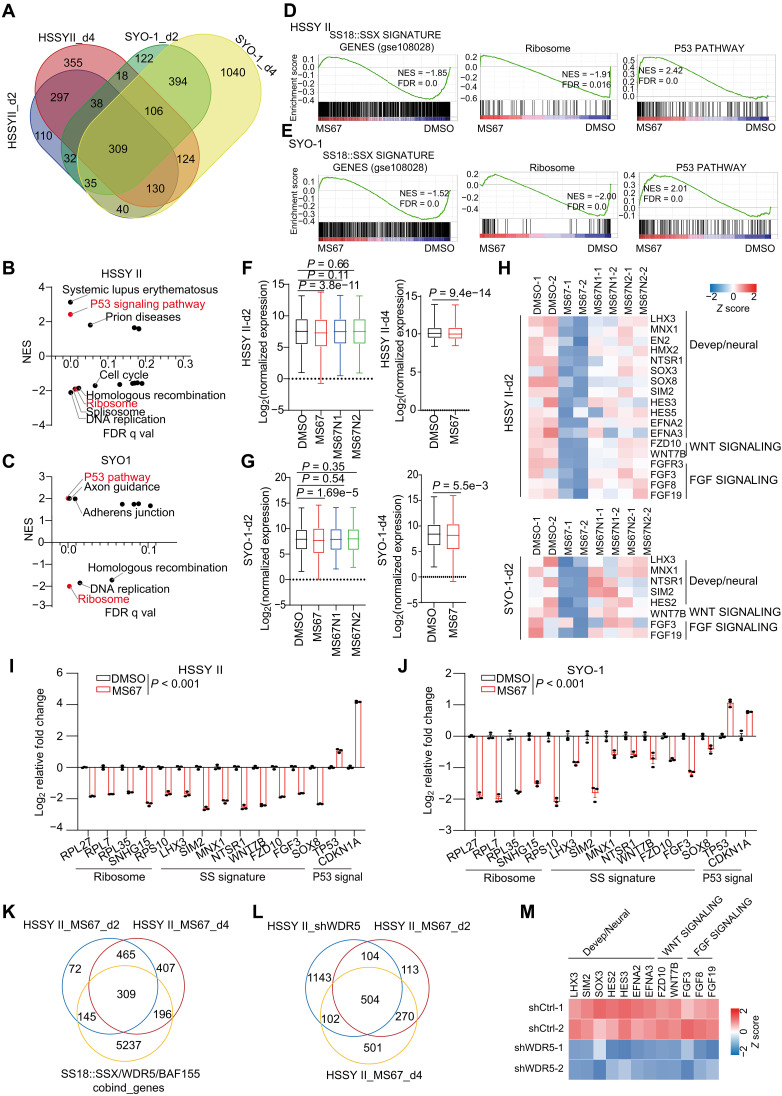
WDR5 PROTAC inhibits transcription of the SS18::SSX-targeted oncogenes and RP-coding genes, leading to P53 activation. (**A**) Venn diagram of DEGs in HSSY II or SYO-1 cells, down-regulated after treatment with 2.5 μM of MS67 versus DMSO for 2 or 4 days (d2 or d4). DEG is defined by a cutoff of fold-change (FC) more than 1.50 and *P*-adj less than 0.05. (**B** to **E**) Summary plot of GSEA [(B) and (C)] and the example enrichment for indicated pathways [(D) and (E)] in HSSY II or SYO-1 cells. The SS18::SSX-associated signature genes were defined to be those down-regulated significantly after SS18::SSX KD in HSSY II cells, with a cutoff of log_2_ value of FC less than −1 and *P*-adj less than 0.001 using a dataset published in (*19*). (**F** and **G**) Box plots showing overall expression of the SS18::SSX signature genes, with Wilcoxon test used for generating *P* value. (**H**) Heatmap showing down-regulation of the indicated SS oncogenes in HSSY II (top) and SYO-1 cells (bottom), using the d2 treatment dataset. (**I** and **J**) RT-qPCR for the indicated gene in HSSY II (I) or SYO-1 cells (J), treated with 2.5 μM of DMSO or MS67 for 2 days (*n* = 3; means ± SEM). *P* values were calculated with two-tail Student’s *t* test. (**K**) Venn diagram using MS67–down-regulated DEGs using the d2 and d4 treatment data and genes cobound by WDR5, SS18::SSX, and BAF155 in HSSY II cells. (**L**) Venn diagram using DEGs in HSSY II cells down-regulated because of stable transduction of shWDR5 versus control (HSSY II_shWDR5) or treatment of MS67 versus DMSO for 2 or 4 days (HSSY II_MS67_d2 or HSSY II_MS67_d4). (**M**) Heatmap showing down-regulation of the indicated SS oncogenes in HSSY II cells, stably transduced with the control (shCtrl-1 and shCtrl-2) or WDR5-targeting shRNA (shWDR5-1 or shWDR5-2). NES, normalized enrichment score; FDR, false discovery rate.

Having defined the effects of WDR5 PROTAC on genome-wide binding of WDR5, the SS18::SSX-containing chromatin remodelers, and the MLL-WDR5 complex-catalyzed H3K4me2/3, we next assessed its effects on the transcriptome of SS cells. Toward this end, we conducted RNA sequencing (RNA-seq) in HSSY II and SYO-1 cells posttreatment with the compound and found that, compared with DMSO controls, both a 2-day and a 4-day treatment with MS67 led to the dramatic changes in gene expression, with more genes down-regulated than up-regulated (fig. S6, A and B, and table S1 to S2; see panels of MS67). Also, there was a substantial overlap between differentially expressed genes (DEGs) identified in the two different SS cell lines, and between the DEGs defined at different treatment time points (day 2 or 4) posttreatment with MS67 versus mock (Fig. 5A). In contrast to the observed dramatic transcriptome-modulatory effect of MS67, there was a general lack of effect by the comparable treatment of MS67N1 or MS67N2 on the transcriptome (fig. S6A and table S1 to S2; see panels of MS67N1 and MS67N2). GO analyses (fig. S6, C and D) and gene set enrichment analysis (GSEA) (Fig. 5, B to E) revealed the MS67 treatment to be associated with down-regulation of the SS18::SSX target genes, RP genes, and developmental genes, as well as the activation of tumor suppressor protein p53. Indeed, closer examination of RNA-seq profiles of both HSSY II and SYO-1 cells after the treatment with MS67 versus mock showed the significantly suppressed expression of the previously-defined SS18::SSX signature genes [based on a previous study (*19*)] in HSSY II or SYO-1 cells, an effect not seen with MS67N1 or MS67N2 (Fig. 5, F and G). Clearly, a suite of developmental and neural genes, WNT signaling and fibroblast growth factor signaling genes, all of which were reported to be involved in SS oncogenesis (*12*, *41*–*43*), were significantly suppressed after the MS67 treatment versus mock (Fig. 5H). Reverse transcription quantitative real-time polymerase chain reaction (RT-qPCR) confirmed down-regulation of the select SS-related signature oncogenes and RP genes, as well as a concurrent activation of P53 and its target, *cyclin-dependent kinase inhibitor 1A* (*CDKN1A*, aka, P21), posttreatment of MS67 versus DMSO in HSSY II (Fig. 5I) or SYO-1 cells (Fig. 5J).

The genes cobound by WDR5, SS18::SSX, and SMARCC1/BAF155 in HSSY II cells exhibited a significant overlap with the MS67–down-regulated DEGs in same cells, thus defining a suite of WDR5- and SS18::SSX-cotargeted genes whose expression is inhibited upon WDR5 degradation by MS67 (Fig. 5K). GO analyses revealed these MS67–down-regulated direct target genes of both WDR5 and SS18::SSX to be most enriched for the transcripts related to RPs, cell cycle progression, development, and P53-related responses (fig. S6E).

To substantiate the on-target effect of MS67, we additionally used the WDR5-targeting shRNA to induce WDR5 KD in HSSY II cells (fig. S1C) and performed RNA-seq (table S3). Venn diagram showed a substantial overlap between DEGs down-regulated by the genetic approach (a WDR5-targeting shRNA) and those by the pharmacologic approach (WDR5-targeting MS67), when compared to their respective controls (Fig. 5L). GSEA and heatmap analyses showed the effects of WDR5 KD to be highly consistent to those of the MS67 treatment—WDR5 levels are positively correlated with the high expression of SS signature genes (fig. S6F, top, and Fig. 5M) and RP genes (fig. S6F, bottom), as well as the suppression of P53 signaling (fig. S6F, middle).

Furthermore, we asked whether there exists a common effect of WDR5 PROTAC among different cancer types. Here, we found that a minority (about 8 to 10%) of DEGs down-regulated by the MS67 treatment in the HSSY II SS cells exhibited similar changes in the MS67-treated MV4;11 acute leukemia cells and MIAPaCa-2 pancreatic cancer cells (fig. S6G) (*26*). GO of these commonly altered transcripts revealed a most enrichment for RP genes (fig. S6H).

Together, treatment of SS cells with the WDR5-targeting PROTAC causes down-regulation of the SS18:SSX-related signature oncogenes. In addition to such a disease-specific effect, the WDR5 PROTAC elicits a common effect seen in different cancer models, that is, RP deregulation and P53 activation. Furthermore, these effects are not seen with the matched non-PROTAC analogs, supporting a notion that WDR5 degradation is required for efficiently suppressing oncogenic nodes in SS.

### WDR5 loss suppresses SS growth at least partly through the RP loss–induced activation of nucleolar stress response and the P53 pathway

**Fig. 6.**
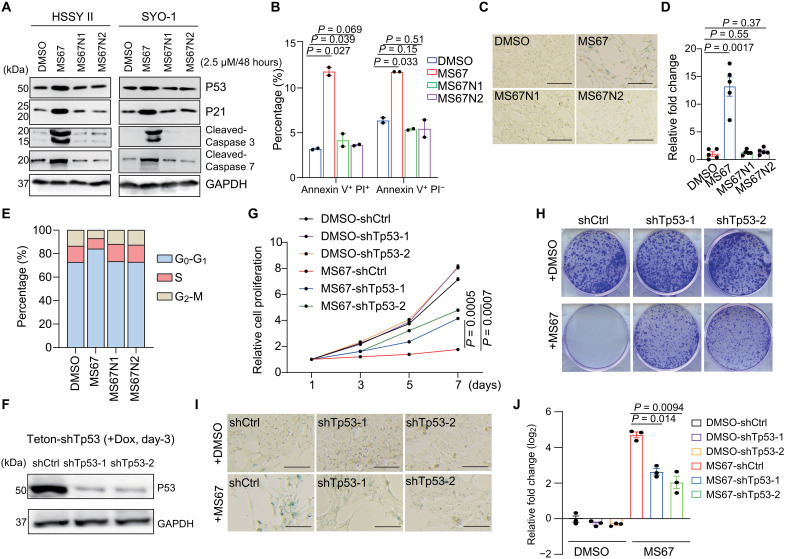
WDR5 PROTAC induces SS cell growth arrest, senescence, and apoptosis at least partly through the P53 signaling activation. (**A**) Immunoblotting of the indicated protein in HSSY II (left) and SYO-1 cells (right), treated with 2.5 μM of DMSO, MS67, MS67N1, or MS67N2 for 2 days. (**B**) Summary of the fluorescence-activated cell sorting (FACS)–based quantification of apoptotic cells (annexin V^+^ and PI^+^) in HSSY II (left) and SYO-1 cells (right), treated with 2.5 μM of DMSO, MS67, MS67N1, or MS67N2 for 2 days (*n* = 2 biological replicates). (**C** to **E**) Representative β-galactosidase (β-Gal) staining images [scale bar, (C) 50 μm], quantification of cells positive for β-Gal staining (D), and quantification of the indicated cell cycle stage (E) in HSSY II cells, treated with 2.5 μM of DMSO, MS67, MS67N1, or MS67N2 for 2 days. (**F**) Immunoblotting of the indicated protein in HSSY II cells, either mock-treated (shCtrl) or stably transduced with a TP53-targeting shRNA (shTp53-1 or shTp53-1), after a 3-day treatment with doxycycline (+Dox) to induce shRNA expression. (**G** and **H**) Cell proliferation plot (G) and colony formation (H) using the indicated shRNA-transduced HSSY II cells, treated with 2.5 μM of DMSO or MS67 (*n* = 3 biological replicates). (**I** and **J**), Representative β-Gal staining images [scale bar, (I) 50 μm] and quantification (J) of cells positive for β-Gal staining (J) in cultures of the indicated shRNA-transduced HSSY II cells, treated with 2.5 μM of DMSO or MS67 for 2 days (*n* = 3 biological replicates). All the experiments were repeated at least twice, with representative results shown here. For all relevant figures, data are represented as means ± SEM. *P* values were calculated with two tail Student’s *t* test.

Previously, it has been reported that P53 responds to the perturbation in ribosomal biogenesis, and, upon a nucleolar stress, P53 is activated to cause the cell cycle arrest, senescence, and/or cell death (*44*, *45*). Compared with mock treatment or the two non-PROTAC negative controls (MS67N1 and MS67N2), only MS67 efficiently activated the P53 pathway as assessed by WB of P53 and P21/CDKN1A, as well as the apoptotic markers, cleaved caspases (Fig. 6A); only MS67 led to apoptosis (Fig. 6B and fig. S7A), senescence (Fig. 6, C and D), and the cell cycle progression arrest (Fig. 6E and fig. S7B). To further test whether the observed P53 activation is responsible for the MS67-induced killing effect, we used either one of the two independent p53-targeting shRNAs to suppress the P53 activation in HSSY II cells (Fig. 6F) and found that such P53 blockade partially but significantly reversed the MS67-elicited effects, including the growth inhibition (Fig. 6G), the decreased colony formation (Fig. 6H), and the induced senescence (Fig. 6, I and J). Overall, the WDR5 loss–induced RP deregulation and nucleolar stress result in the activation of P53 pathway, contributing to the SS growth suppression.

### WDR5 PROTAC suppresses the malignant growth of SS in vivo

**Fig. 7.**
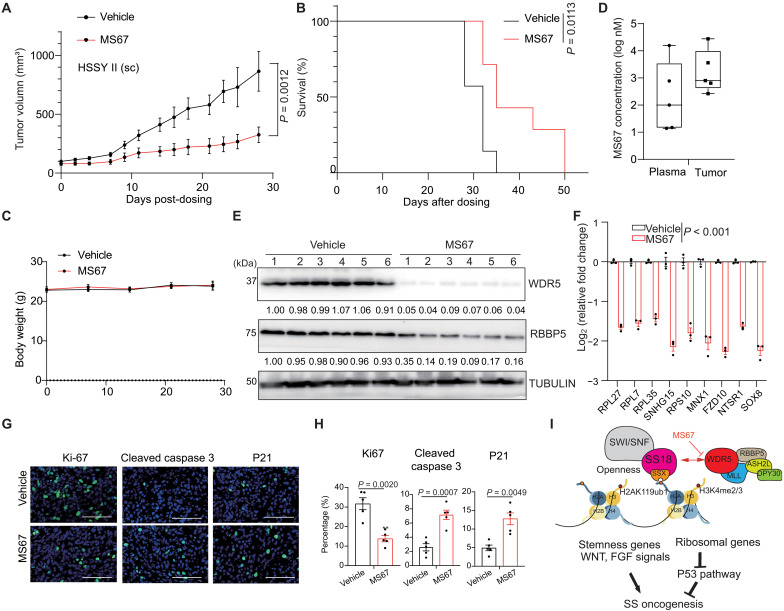
WDR5 PROTAC suppresses SS oncogenesis in vivo. (**A** to **C**) Growth of HSSY II CDX in NSG mice via subcutaneous (sc) inoculation [(A) means ± SEM in *y* axis], Kaplan-Meier survival curve (B), and averaged body weight of mice (C), treated with vehicle (*n* = 7) or MS67 (*n* = 7; a dose of 75 mg/kg, twice daily via intraperitoneal injection for 5 days per week). Statistical analysis for tumor growth was performed using two-way analysis of variance (ANOVA) followed by Sidak’s multiple comparison test, with statistical calculation at the last dosing time point labeled, while statistical analysis of survival was conducted by log-rank (Mantel-Cox) test. (**D** to **F**) Mass spectrometry–based measurement of MS67 concentration in the plasma and tumor samples (D), and WB (E) and RT-qPCR (F) for the indicated factor in tumors, collected 2 hours after the last dosing from vehicle- or MS67-treated mice. The numbers under gel images in (E) show the relative protein levels normalized to the Vehicle#1 sample. *Y* axis in (F) shows fold change of expression in the tumors from the MS67-treated versus vehicle-treated mice (*n* = 3, means ± SEM). The two-tailed Student’s *t* test was used to calculate *P* value. (**G** and **H**) Representative IF images [scale bar, (G) 50 μm] and quantification of the percentage of cells positive for the indicated marker (H) in tissue sections of tumors, isolated from vehicle- or MS67-treated mice (*n* = 5, means ± SEM). The two-tailed Student’s *t* test was used to calculate *P* value. (**I**) A model that the WDR5-containing KMT2/MLL complexes and the SS18::SSX-harboring SWI/SNF chromatin-remodeling complexes interact within the condensates and colocalize genome-wide, which operate to maintain both the openness and high H3K4 methylation levels at their target chromatin (demarcated by H2AK119ub, a histone modification recognized by a SSX segment in SS18::SSX), enforcing an oncogenic gene-expression program and SS pathogenesis.

Last, we tested the effect of WDR5-targeting PROTAC on SS oncogenesis in vivo. Here, we generated an HSSY II CDX model in immunodeficient mice nonobese diabetic (NOD)–scid IL2Rgamma^null^ (NSG) mice and found that, compared to vehicle, MS67 effectively reduced the CDX growth in vivo (Fig. 7A) and significantly prolonged the survival of SS-bearing animals (Fig. 7B). In addition, MS67 did not cause the obvious changes in the body weight, suggesting a lack of general toxicity (Fig. 7C). Average concentration of MS67 in the plasma and CDX samples, which were isolated from mice 2 hours after the last dose of MS67, was measured to be approximately 3 and 6 μM, respectively (Fig. 7D), which reaches the EC_50_ value measured in vitro (fig. S1G). Using the collected CDX samples, we verified the MS67-induced WDR5 and RBBP5 degradation (Fig. 7E). In the CDX samples collected from MS67- versus mock-treated mice, we also observed the significantly decreased expression of WDR5 target genes including the RP genes (such as RPL27, RPL7, and RPL35) and the SS oncogenes (such as FZD10 and SOX8) (Fig. 7F), as well as the decrease of the proliferation marker (Ki-67) and concurrent increase of the cleaved caspase 3 and P21 (Fig. 7, G and H), demonstrating the in vivo effects of MS67 dosing. Together, WDR5 is a critical SS dependency, as demonstrated in both the in vitro and in vivo settings, and targeting WDR5 by PROTAC represents a promising SS therapeutic (see a model in Fig. 7I).

## DISCUSSION

Human cancers, SS included (*1*), recurrently target a range of chromatin pathway genes, pointing to a causal and central role of epigenetic lesions in driving the oncogenesis and disease progression (*46*–*50*). Compared with genetic alterations, epigenetic aberrations are potentially reversible, allowing those malignant cell populations to regain the normal cell states (*47*, *49*, *51*). With the advent of drugs targeting specific epigenetic pathways, harnessing the cancer-related epi-targets emerges as an attractive and promising therapeutic strategy (*47*, *49*, *52*, *53*).

SS is a rare yet aggressive cancer type characterized by an aberrant chromosomal translocation, SS18::SSX (*6*, *7*). Development of the ways to block SS18::SSX’s oncogenic activities holds a great promise for improving the current treatment of SS. In this work, we show that there exists an unexplored cross-talk between WDR5 and the SS18::SSX-containing chromatin-remodeling complexes in SS—Our integrated approaches covering co-IF, co-IP, and genome-wide profiling (RNA-seq, CUT&Tag, and CUT&RUN) demonstrated that the two physically associate with one another, coexist in the nucleoplasmic puncta reminiscent of the phase-separated onco-condensates (*54*), and also significantly colocalize genome-wide. It is worth mentioning that we did not observe similar interaction between the KMT2/MLL-WDR5 complexes and the SWI/SNF complexes in U2OS cells, an SS18::SSX-negative non-SS cell line (Fig. 2B versus Fig. 2C), suggesting an SS18::SSX-dependent association between the two complexes. Such an SS-unique interaction can explain a quite selective killing effect by WDR5-degrading PROTAC on SS cells when compared to non-SS sarcoma cells (Fig. 1E versus fig. S1E). Second, we used both genetic manipulation and chemical biology approaches and showed WDR5 to be crucial for sustaining the SS18::SSX-associated transcriptomic program, which is enriched for transcripts related to SS proliferation and stemness (as illustrated in a model of Fig. 7I). In addition, a common effect of WDR5 depletion across different cancer models (covering SS, leukemia, and pancreatic cancer) (*26*) is deregulation of RP-coding genes, leading to activation of P53 signaling, consistent with prior studies using other tumor models (*26*, *39*, *40*). While WDR5 degradation by PROTAC does not substantially affect H2AK119ub at the SS18::SSX target sites, the WDR5 PROTAC decreases the overall binding of both SS18::SSX and the SS18::SSX-associated SWI/SNF complexes (such as SMARCC1/BAF155), with the effect on the latter seemingly stronger than that on SS18::SSX. These observations, together with previous reports (*18*, *19*, *21*, *22*), suggest a model that an H2AK119ub-reading activity harbored within the SSX segment of SS18::SSX may direct the initial recruitment of onco-fusion to the Polycomb complexes–targeted genomic sites (*21*, *22*) where the onco-fusion further assembles the SWI/SNF complexes and recruits the KMT2/MLL-WDR5 complexes as well, which in turn, act in concert to modulate the local chromatin landscape in a synergistic fashion, leading to gene activation and SS formation. In addition, a seemingly stronger effect of MS67 treatment on the overall chromatin binding of SMARCC1/BAF155 (when compared to chromatin association of SS18::SSX) is consistent with this model, which also indicates that SS18::SSX’s initial loading onto chromatin and the events of recruitment and/or spreading of other key players (namely, the WDR5-containing KMT2/MLL complexes and the SS18::SSX-containing SWI/SNF complexes) may be separated events. The details of such an SS18::SSX-directed signaling merit additional investigation. Third, we demonstrated the pharmacological degradation of WDR5 by PROTAC to be superior to the match WDR5 PPI inhibitor for the SS treatment, and the tumor-killing effects by MS67 are much more potent in the SS18::SSX-positive SS cells, when compared to non-SS sarcoma cells that do not carry SS18::SSX. SS exhibits a preferential WDR5 dependency, and WDR5 represents a valuable therapeutic target in SS. Last, a framework established in this study for identifying epigenetic dependency in SS and developing the mechanism-based epigenetic therapies is applicable to other incurable cancers. We remain optimistic about significant progress along these lines of research in the years to come.

### Limitation of the study

In the future, research effort shall be directed to better understand the molecular mechanism underlying the association between SS18::SSX and WDR5 in a context of puncta. Furthermore, WDR5-targeted therapies shall be tested in the clinically relevant models of SS, either using WDR5 PROTAC alone or in combination with the existing anti-SS agents.

## MATERIALS AND METHODS

### Ethical approval and usage of animals

All animal procedures were performed in accordance with the protocols approved by Institutional Animal Care and Use Committee (IACUC) of Duke University (protocol # A254-23-12). The NSG mice were obtained from the Jackson Laboratory and housed in the standard specific pathogen–free facility. For the in vivo efficacy studies, the subcutaneous inoculation of HSSY II cells was performed to establish the CDX. Group sizes were selected on the basis of prior knowledge. The mice were matched for age, gender, and genetic background and randomized appropriately, but blinding was not applied. Briefly, 5 million HSSY II cells were mixed with the Matrigel (Corning, catalog no. 354248) in a volume of 200 μl and injected subcutaneously to both flanks of each one of 8-week-old NSG mice. The mice were randomly subgrouped to the vehicle or compound treatment cohort when the average CDX tumor size reaches 100 mm^3^, followed by the treatment with vehicle or MS67. For the in vivo studies, MS67 (in its HCl salt form) was dissolved in a solution formulation of 5% N-Methyl-2-Pyrrolidone (NMP), 5% solutol HS-15, and 90% normal saline as we described before (*26*). The used dose of MS67 was 75 mg/kg, twice daily via intraperitoneal injection for 5 days per week (from Monday to Friday). Tumor volume was recorded via caliper every 2 to 3 days. The inclusion and exclusion criteria were based on the IACUC protocol, and the study was terminated when the tumor size reached the IACUC allowed limit, or the body weight loss is greater than 15%.

### Public datasets

The gene depletion effect scores were generated using the publicly accessible datasets of the Cancer Dependency Map (DepMap; https://depmap.org/portal/) DEMETER2 (RNAi) screening datasets (*55*, *56*). This work focuses solely on epigenetic regulators, with the gene list and the extracted gene depletion effect scores available upon request. A previously published transcriptomic dataset of HSSY II cells [under the National Center for Biotechnology Information (NCBI) Gene Expression Omnibus (GEO) accession number GSE108028 (*19*)] was used to define the SS18::SSX signature genes as those significantly down-regulated transcripts upon the KD of SS18::SSX versus mock by using a cut-off of log_2_ value of fold change less than −1 and adjusted *P* (*P*-adj) value less than 0.001.

### Cell culture

The human SS patient–derived cell lines, HSSY II and Yamato-SS, were acquired from the RIKEN BioResource Research Center cell bank. SYO-1 and Mojo cell lines were shared by G Schwartz (Columbia University) (*57*) and M. Ladanyi (Memorial Sloan Kettering Cancer Center). Other cells used in this work were obtained from American Type Culture Collection and include U2OS (HTB-96), A-673 (CRL-1598), RD-ES (HTB-166), A204 (HTB-82), 293T (CRL-3216), and NIH-3 T3 (CRL-1658). Rh4 and Rh10 cells were shared by Y. Diao (Duke University). For cell culture, the high-glucose Dulbecco’s modified Eagle’s base medium (Gibco) supplemented with fetal bovine serum and antibiotics were used following the vendor’s protocols. The lack of mycoplasma contamination was confirmed routinely using the commercial detection kits (Lonza, LT27-286).

### Bioanalysis of MS67 in mouse plasma and tumor samples

HSSY II cells were injected into NSG mice as described above, and the bioanalysis of MS67 in mouse plasma and tumor samples was performed as previously described (*26*). Briefly, tumor and plasma samples were collected at 2 hours after the last dose. For plasma preparation, 200 μl of blood was collected in an Eppendorf tube pretreated with EDTA. Samples were centrifuged at 3000 rpm for 10 min at 4°C, and the supernatant was collected and stored at −80°C. Tumors were harvested immediately after the animals were euthanized and then snap frozen in liquid nitrogen and stored at −80°C.

### Chemicals

OICR-9429, MS67, and MS67N1 (previously named as MS67N) were synthesized, followed by validation of chemical identity and purity as previously described (*26*). MS67N2 is synthesized following the general chemistry methods as previously described (*26*) and the synthetic route for its preparation as well as its chemical identity and purity validation data are provided in the raw image file. Ten millimolar of compound stock was prepared by dissolving in DMSO (MilliporeSigma, D2650) and used in the in vitro assays.

### Cell growth and colony formation assays

For proliferation assays, the cells were seeded in triplet in the 96-well plates at a density of 1000 cells per well. On the next day, the cells were treated with vehicle or a range of tested concentration of MS67, MS67N1, MS67N2, or OICR-9429. The medium was refreshed every 48 hours to maintain drug concentration. MTS assay was performed with the CellTiter 96 AQueous One Solution Cell Proliferation Assay kit (Promega, G3582) based on the vendor’s protocol. For the colony formation assays, the cells were seeded in the six-well plates at a density of 2000 cells per well. On day 7, the plates were washed in phosphate-buffered saline (PBS) and stained with crystal violet after fixation using methanol for 10 min.

### Plasmids

The pLKO1-puro vector was obtained from Addgene (#10878). The pLKO1-shWDR5-1, pLKO1-shWDR5-2, pLKO1-shSSX-1, and pLKO1-shSSX-2 were generated by insertion of the previously validated WDR5-targeting (*26*) or SS18::SSX-targeting shRNAs (*18*) to the EcoRI and AgeI restriction enzyme sites of pLKO1 vector. The doxycycline-inducible shRNA expression vector (pLKO-Teton-puro) for TP53 targeting (pLKO-Teton-puro-shTP53-1 and pLKO-Teton-puro-shTP53-2) (*58*) were gifts from J. Morris (UNC). Primers used for plasmid construction are listed in the Supplementary table.

### Gene KD

The pLKO1-puro–based lentiviral plasmids, which contain either control shRNAs (pLKO-puro-shGFP or doxycycline-inducible pLKO-Teton-puro-shCtrl) or the independent shRNAs for knocking down the gene of interest, were transfected together with the packaging plasmids to 293T cells for lentivirus production. The collected lentiviral preparation was used to infect cells and generate stable cell lines as before (*59*). Doxycycline (1 μg/ml) was used for inducing TP53 KD in the stable cell lines as described before (*58*).

### Western blot

The total cell lysate sample was generated by boiling the PBS-rinsed cells directly in the SDS protein sample buffer, followed by WB as previously described (*60*). The primary antibodies used in the study (all diluted at 1:1000) include those against WDR5 (Cell Signaling Technology, 13105), WDR5 (Santa Cruz Biotechnology, sc-393080), SS18::SSX (Cell Signaling Technology, 72364), SS18 (Cell Signaling Technology, 21792), SMARCC1/BAF155 (Cell Signaling Technology, 11956), SMARCA4/BRG1 (Abcam, ab110641), KMT2A/MLL1 (Cell Signaling Technology, 14197), RBBP5 (Cell Signaling Technology, 13171), H3K4me1 (Abcam, ab8895), H3K4me2 (Cell Signaling Technology, 9725), H3K4me3 (Cell Signaling Technology, 9751), general H3 (Abcam, ab1791), cMYC (Abcam, ab32072), P53 (Cell Signaling Technology, 2524), P21 (Cell Signaling Technology, 2947), cleaved caspase 3 (Cell Signaling Technology, 9661), cleaved caspase 7 (Cell Signaling Technology, 8438), glyceraldehyde-3-phosphate dehydrogenase (Cell Signaling Technology, 2118), and tubulin (Cell Signaling Technology, 2146). The images of immunoblots were taken using ImageQuant 800 (Cytiva) following the vendor’s protocol.

### Coimmunoprecipitation

Protein A/G magnetic beads (Bio-Rad) were used for co-IP following the manufacturer’s protocol. Briefly, cells grown in a 15-cm plate were collected, rinsed twice with cold PBS, and lysed on ice using the lysis buffer (50 mM Tris-Cl pH 7.5, 150 mM NaCl, 2 mM MgCl2, 0.5% NP-40, 10% glycerol, with the protease inhibitor cocktail freshly added). Total cellular protein sample was extracted at 4°C for 30 min with rotation, followed by centrifugation at 13,000 rpm for 30 min. The antibody of WDR5 (Cell Signaling Technology, 13105) or SS18::SSX (Cell Signaling Technology, 72364) or nonspecific rabbit IgG (Cell Signaling Technology, 2729) was added and incubated with lysates overnight at 4°C, followed by the addition of 20 μl of protein A/G magnetic beads (Bio-Rad) and incubation for 6 hours at 4°C with rotation. Last, the beads were collected and rinsed, and the bound proteins were eluted off the beads in 50 μl of the SDS sample buffer per IP after heating at 90°C for 15 min. The samples were collected and loaded onto SDS–polyacrylamide gel electrophoresis gels for WB.

### Chromatin fractionation

The cells were collected, washed twice with cold PBS, and resuspended in 200 μl of the CSK buffer [10 mM Pipes (pH 7.0), 300 mM sucrose, 300 mM NaCl, 3 mM MgCl_2_, and 0.1% of Triton X-100; with the protease inhibitor cocktail freshly added], followed by incubation on ice for 30 min. Next, the sample was subject to centrifugation at 1300*g* for 5 min at 4°C to collect the supernatant (which represents the soluble fraction) and pellet (which represents the chromatin-associated fraction). The latter cell pellet was dissolved in the 1× SDS loading buffer by heating before use.

### IF and immunohistology staining

The antibodies used for IF studies of cells or tissue sections include WDR5 (Santa Cruz Biotechnology, sc-393080, 1:500), SS18::SSX (Cell Signaling Technology, 72364, 1:500), cleaved caspase3 (Cell Signaling Technology, 9661, 1:1000), Ki-67 (Abcam, ab15580, 1:1,000), and P21 (Cell Signaling Technology, 2947, 1:500). IF of the cultured cells was conducted as before (*61*). Tumor samples were collected freshly and embedded in OCT compound (Tissue Tek), followed by the cryosection preparation using a Leica cryostat equipment for the subsequent histological studies. The β-galactosidase staining was performed with a kit of Cell Signaling Technology (catalog no. 9860) based on the vendor’s instruction. Images were taken using the Zeiss 880 Airyscan confocal (Zeiss) and analyzed by ZEN software.

### FACS analysis

The single-cell suspensions were prepared in cold PBS, stained, and subject for fluorescence-activated cell sorting (FACS) analysis using a Cytek Aurora System (Cytek Biosciences). Apoptosis analysis was conducted by using the Annexin V-FITC Apoptosis Staining and Detection Kit (Abcam, ab14085) following vendor’s instructions. For cell cycle progression analysis, the propidium iodide nucleic acid stain kit (Invitrogen, P3566) was used following manufacturer’s protocol. FACS data were analyzed with FlowJo 7.6 software.

### CUT&Tag, CUT&RUN, and data analysis

CUT&Tag (*33*) and CUT&RUN (*35*) were performed as we previously described (*59*, *62*–*64*) by using commercial kits from EpiCypher and following the manufacturer’s detailed protocols. The used antibodies include WDR5 (Cell Signaling Technology, 13105), SMARCC1/BAF155 (rabbit, Cell Signaling Technology, 11956), SS18::SSX (Cell Signaling Technology, 72364), green fluorescent protein (GFP, Abcam, ab290), H3K4me2 (Cell Signaling Technology, 9725), H3K4me3 (Cell Signaling Technology, 9751), and H2AK119ub1 (Cell Signaling Technology, 8240S). For CUT&Tag or CUT&RUN of SS18::SSX, the human SS cells were added with 5% of the NIH-3 T3 murine embryonic fibroblast cells (stably transduced with H2B-GFP) across all samples as a spike-in control, and 1 μl of anti-SS18::SSX primary antibody plus 1 μl of anti-GFP primary antibody was used; for the rest of CUT&Tag and CUT&RUN assays, 1 μl of primary antibody, which recognizes the protein of interest in both human and mouse cells (such as WDR5, BAF155, BRG1, or histone mark), was applied to the same sample containing a mixture of human SS cells and 5% of the above murine fibroblasts, with the latter used as a spike-in control for signal normalization.

We followed the same CUT&Tag protocol as before (*33*, *63*) for sample preparation. In brief, the cell pellet was washed with the wash buffer [20 mM Hepes (pH 7.5), 150 mM NaCl, 0.5 mM spermidine, and 1× protease inhibitor cocktail], and 10 μl of the concanavalin A–coated magnetic beads was added {Bangs Laboratories catalog no. BP531; first activated in beads activation buffer [20 mM Hepes (pH 7.9), 10 mM KCl, 1 mM CaCl_2_, and 1 mM MnCl_2_]}, followed by incubation at room temperature (RT) for 10 min. After removing the unbound supernatant, the bead-bound cells were resuspended in the Digitonin 150 buffer [20 mM Hepes (pH 7.5), 150 mM NaCl, 0.5 mM spermidine, 1× protease inhibitor cocktail, and 0.01% Digitonin] containing 2 mM EDTA and the appropriate primary antibody as described above. The antibody incubation was performed on a rotating platform overnight at 4°C. The next day, the sample was incubated with the appropriate secondary antibody (diluted 1:50 in the Digitonin 150 buffer) at RT for 30 min. The cells were then washed using a magnet stand with the Digitonin 150 buffer to remove the free antibodies. A 1:200 dilution of pA-Tn5 adapter complex was prepared in the Digitonin 300 buffer [0.01% Digitonin, 20 mM Hepes (pH 7.5), 300 mM NaCl, 0.5 mM spermidine, and 1× protease inhibitor cocktail] and added to the sample, followed by incubation at RT for 1 hour. The samples were washed again in the Digitonin 300 buffer to remove the unbound pA-Tn5 protein. Next, the cells were resuspended in the tagmentation buffer (10 mM MgCl_2_ in the Digitonin 300 buffer) and incubated at 37°C for 1 hour. To stop the tagmentation, 2.25 μl of 0.5 M EDTA, 2.75 μl of 10% SDS, and 0.5 μl of Proteinase K (20 mg/ml) was added to 50 μl of sample, which was incubated at 55°C for 30 min, and then at 70°C for 20 min to inactivate Proteinase K. DNA was purified with Ampure XP beads following the manufacturer’s instruction and eluted in the elution buffer [10 mM tris (pH 8.0)]. To generate multiplexed libraries, the eluted DNA was mixed with a universal i5 primer, a barcoded i7 primer (with a unique barcode used for each sample), and 2× PCR master mix. A post-PCR clean-up step was performed using 0.9× volume of Ampure XP beads, and libraries were eluted in 30 μl of the elution buffer [10 mM tris (pH 8.0)].

CUT&RUN was conducted as we described (*59*, *62*, *64*). Briefly, the cells were washed and immobilized onto activated concanavalin A magnetic beads as described above, followed by incubation at RT for 10 min, permeabilization, and then incubation with the primary antibody on nutator overnight at 4°C. On the next day, the cell-bead slurry was washed twice and incubated with pAG-MNase (1:20 dilution, EpiCypher, catalog no. 15-1116) for 10 min at RT, followed by addition of CaCl_2_ and a 2-hour incubation at 4°C for the targeted chromatin cleavage by activated MNase. After chromatin digestion, the stop buffer was added and chromatin fragments were released into the supernatant, followed by purification using the Monarch DNA Cleanup Kit (NEB, catalog no. T1030) per the manufacturer’s instruction. Ten nanograms of purified DNA was subject to library preparation using the NEB Ultra II DNA Library Prep Kit (NEB, catalog no. E7645).

The libraries were subjected to deep sequencing using Illumina NextSeq 2000 equipment. After sequencing, the reads in fastq files were first mapped to the main reference genome (hg38) and to the spike-in control genome (mm10) using Bowtie2 (v.2.4.4). Non-primary alignment and PCR duplicates were removed from aligned data, respectively, by using Samtools (v.1.10) and Picard “MarkDuplicates” function (v.2.18.2). For the normalization against spike-in controls, a scale factor was calculated by comparing the total number of aligned spike-in reads among different sample groups, which was then used for signal normalization (*65*). Peak calling was performed using MACS2 (v.2.2.6) and peak annotations conducted by the HOMER annotatePeaks.pl function. Read densities were visualized at specific gene loci using the Integrative Genomics Viewer (Broad Institute). GO analysis for genes associated with the annotated peaks was generated using annotatePeaks.pl function and DAVID Bioinformatics (https://davidbioinformatics.nih.gov/), while the motif analysis was conducted by the findMotifsGenome.pl function.

### RNA-seq and data analysis

Total RNA was extracted using the RNeasy Plus Mini Kit (Qiagen, 74136), with an on-column DNA digestion step performed to remove the genomic DNA. Equal amount of ERCC RNA Spike-In Mix (Thermo Fisher Scientific, 4456740) was added to all samples as a spike-in control before library preparation, followed by generation of multiplex libraries with the NEBNext Ultra II RNA Library Prep Kit for Illumina (New England BioLabs, E77705) based on the vendor’s standard protocols. The reads in the fastq files of RNA-seq data were aligned as before (*59*), with transcript abundance for each sample generated by using salmon (v.1.4.0). Then, DEGs were defined by DESeq2 (v.1.38.2). GSEA using the Molecular Signatures database (MSigDB) C2 curated gene sets were conducted using GSVA (v.1.30.1) as described before (*59*, *62*, *64*), and the GO analysis was conducted using clusterProfiler R package (*66*).

### Reverse transcription followed by qPCR

Total RNA samples of cells were prepared using the RNeasy Mini Kit (Qiagen) following the vendor’s protocol. For RT-qPCR, reverse transcription was performed first using the total RNA and iScript cDNA Synthesis Kit (Bio-Rad), followed by real-time qPCR with the iTaq universal SYBR green supermix (Bio-Rad) and the Applied Biosystems ViiA 7 system. Primers used for RT-qPCR are listed in the Supplementary table.

### Statistical analysis

Comparisons in qPCR data were performed using an unpaired two-tailed Student’s *t* test. Quantitative data displayed as histograms are shown as means ± SEM. GraphPad Prism 8 software, R, and Excel (Microsoft 2019 version) were used to assess statistical significance. Statistical significance is generally set at a *P* value less than 0.05. *P*-adj values were two-sided, and multiple comparison *P*-adj values calculated by DESeq2. Wilcoxon test was conducted in R, and the survival curve statistical analyses were conducted with the log-rank (Mantel-Cox) tests. Statistical analysis for the tumor growth curves was performed using two-way repeated measures analysis of variance (ANOVA) followed by Sidak’s multiple comparisons test, with the significance at the last time point shown. All experiments were performed with at least two to three replicates and were repeated independently and biologically at least twice with similar results. When possible, we used independent tools and approaches (such as genetic KD and pharmacologic degradation of WDR5, MS67, and MS40 as WDR5 PROTACs, CUT&Tag, CUT&RUN, etc.) to enhance rigor of the study.
