# Supplementary material for: Pharmacologic degradation of WDR5 suppresses oncogenic activities of SS18::SSX and provides a therapeutic of synovial sarcoma
Source: Sci Adv. 2025 Apr 23;11(17):eads7876. doi: 10.1126/sciadv.ads7876 (PMC12017321; doi:10.1126/sciadv.ads7876)
Supplement: Supplementary file 1 — Figs. S1 to S7 Legends for tables S1 to S3 Table S4 [file sciadv.ads7876_sm.pdf]

Science Advances
AAAS

Supplementary Materials for

Pharmacologic degradation of WDR5 suppresses oncogenic activities of SS18::SSX and provides a therapeutic of synovial sarcoma

Yao Yu *et al.*

Corresponding author: Ling Cai, ling.cai@duke.edu; Gang Greg Wang, greg.wang@duke.edu

Sci. Adv. **11**, eads7876 (2025)
DOI: 10.1126/sciadv.ads7876

The PDF file includes:

Figs. S1 to S7
Legends for tables S1 to S3
Table S4

Other Supplementary Material for this manuscript includes the following:

Tables S1 to S3

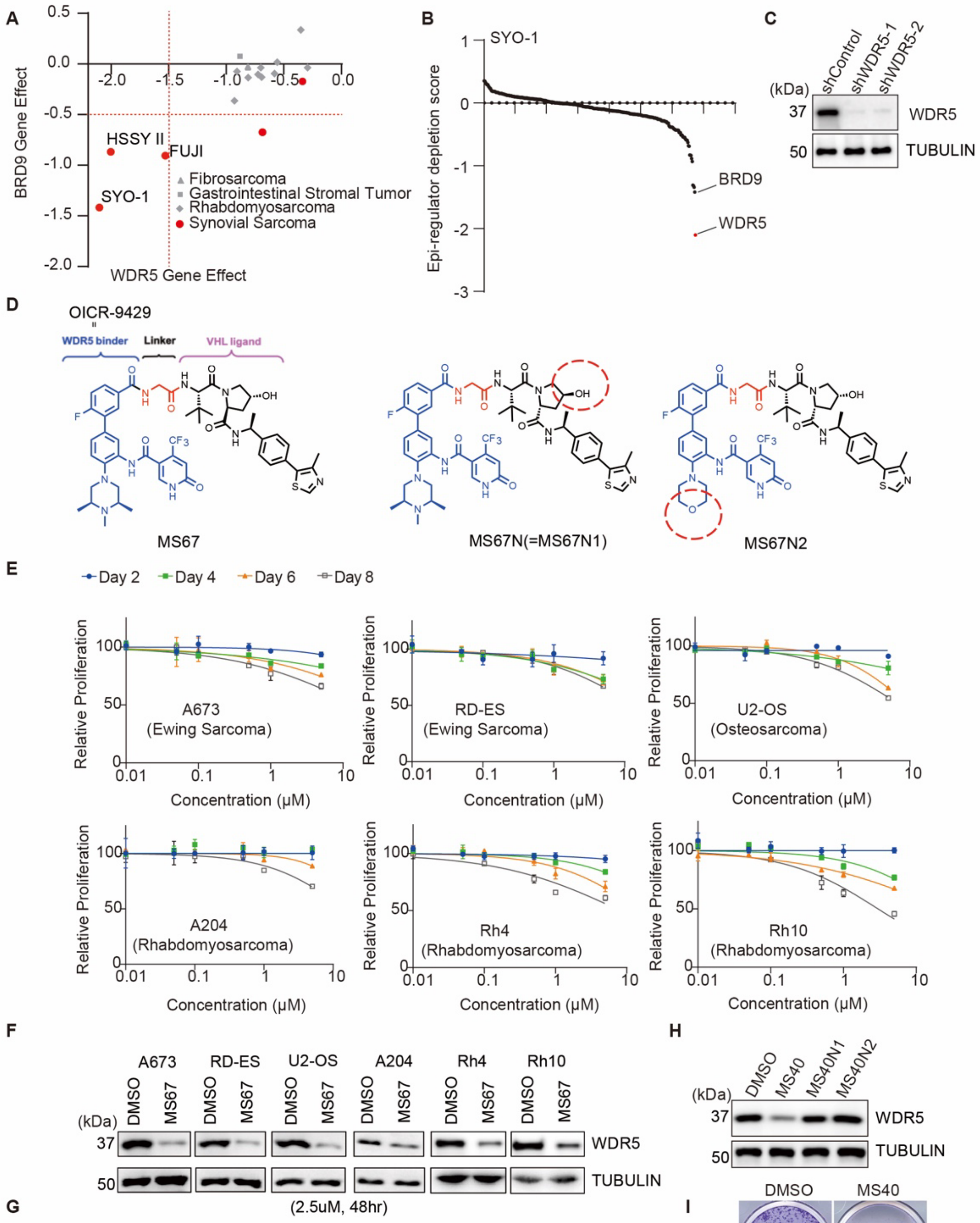

G

	OICR-9429	MS67	MS67N1	MS67N2
HSSY II	>10,000 nM	51 nM	>10,000 nM	>10,000 nM
SYO-1	>10,000 nM	124 nM	>10,000 nM	>10,000 nM
Yamato-SS	>10,000 nM	179 nM	>10,000 nM	>10,000 nM
MoJo	>10,000 nM	134 nM	>10,000 nM	>10,000 nM

I

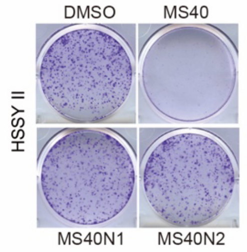

J

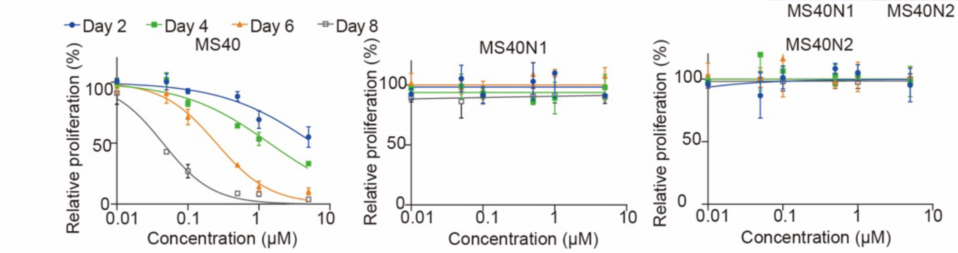

Fig. S1. MS67 potently and selectively inhibits malignant growth of SS cells. (**A**) Depletion scores based on the DepMap DEMETER2 (RNAi) dataset showing the WDR5 (x-axis) and BRD9 (y-axis) dependency in a set of SS (red) and non-SS (gray) cell lines of human sarcomas. Three SS cells (HSSY II, SYO-1 and Fuji) are labeled and exhibit striking dependencies on BRD9 and WDR5 both. (**B**) Depletion scores of all epigenetic regulators in SYO-1 cells based on the DepMap DEMETER2 dataset. (**C**) Western blot (WB) of WDR5 and Tubulin in the HSSY II cells stably transduced with a control shRNA or the shRNA targeting WDR5 (shWDR5-1 or shWDR5-2). (**D**) Chemical structure of the indicated compound. Red circle highlights the modification introduced to either MS67's WDR5 binder moiety (MS67N1) or its VHL ligand moiety (MS67N2) to generate the analog controls. (**E**) Plots of growth inhibition in a cohort of non-SS sarcoma cell lines, treated with various concentration (x-axis) of MS67 for 2, 4, 6 or 8 days. Y-axis, presented in the mean ± SEM of data, shows the relative growth after normalization of the total cell number in compound-treated cultures against the DMSO-treated controls (n = 3 independent experiments). (**F**) WB of WDR5 and Tubulin in the indicated non-SS sarcoma cells, treated with 2.5μM of DMSO or MS67 for 48 hours. (**G**) Summary for the EC50 value of OICR-9429, MS67, MS67N1 or MS67N2 after an eight-day treatment of the indicated cells. (**H**) WB of WDR5 and Tubulin in the HSSY II cells, treated with 2.5μM of DMSO, MS40, MS40N1 or MS40N2 for 48 hours. (**I-J**) Images of colony formation assay (**I;** 2.5μM of compound) and plots of growth inhibition (**J**) using the HSSY II cells. Cells in **J** were treated with the indicated concentration (x-axis) of MS40, MS40N1 or MS40N2 for either 2, 4, 6 or 8 days.

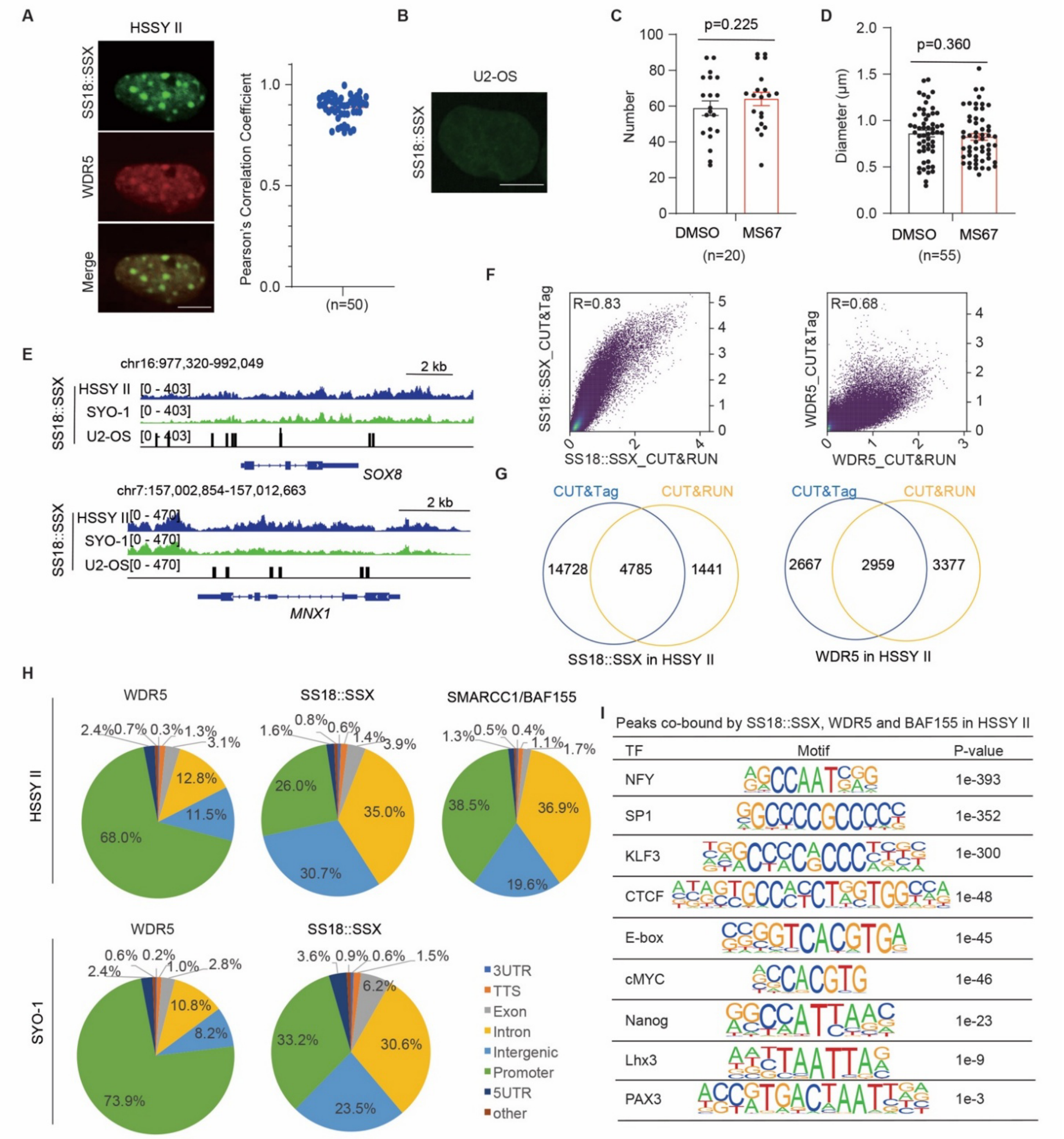

Fig. S2. WDR5 colocalizes with SS18::SSX genome-wide in SS cells. (**A**) Left: Representative immunofluorescence (IF) images of SS18::SSX and WDR5 in HSSY II cells, a SS18::SSX1-positive human SS cell line. Scale bar, 5μm. Right: Pearson's Correlation Coefficient using IF signals of SS18::SSX1 and WDR5 in HSSY II cells (n = 50). (**B**) A representative image of SS18::SSX IF in U2OS cells, an osteosarcoma line lacking SS18::SSX expression. Scale bar, 5μm. (**C-D**) Plots showing the total number (**C**, n=20) and diameter of the SS18::SSX condensates (**D**, n = 55), detected by IF using anti-SS18::SSX antibodies, in HSSY II cells treated with 2.5μM of

DMSO or MS67 for 48 hours. *P* values were calculated with two-tail Student t-test. (**E**) Integrative Genomics Viewer (IGV) tracks showing the SS18::SSX CUT&Tag signals at the indicated gene in HSSY II (SS18::SSX1 positive), SYO-1 (SS18::SSX2 positive) or U2-OS cells (lacking SS18::SSX). (**F**) Pearson correlation coefficient plot using the CUT&Tag and CUT&RUN signals of SS18::SSX (left) or WDR5 (right) in HSSY II cells (mock-treated). (**G**) Venn diagram using the CUT&Tag and CUT&RUN peaks of SS18::SSX (left) or WDR5 (right) in HSSY II cells. Calling of the peaks uses a standard of the -Log10 value of qVal greater than 3 and signalValue greater than 3 (for SS18::SSX) or 6 (for WDR5). (**H**) Pie chart showed the genomic annotation distribution of the called peaks of WDR5, SS18::SSX or BAF155 in HSSY II (top) and SYO-1 cells (bottom). (**I**) Unbiased motif search analysis using the peaks co-bound by SS18::SSX, WDR5 and BAF155 in HSSY II cells.

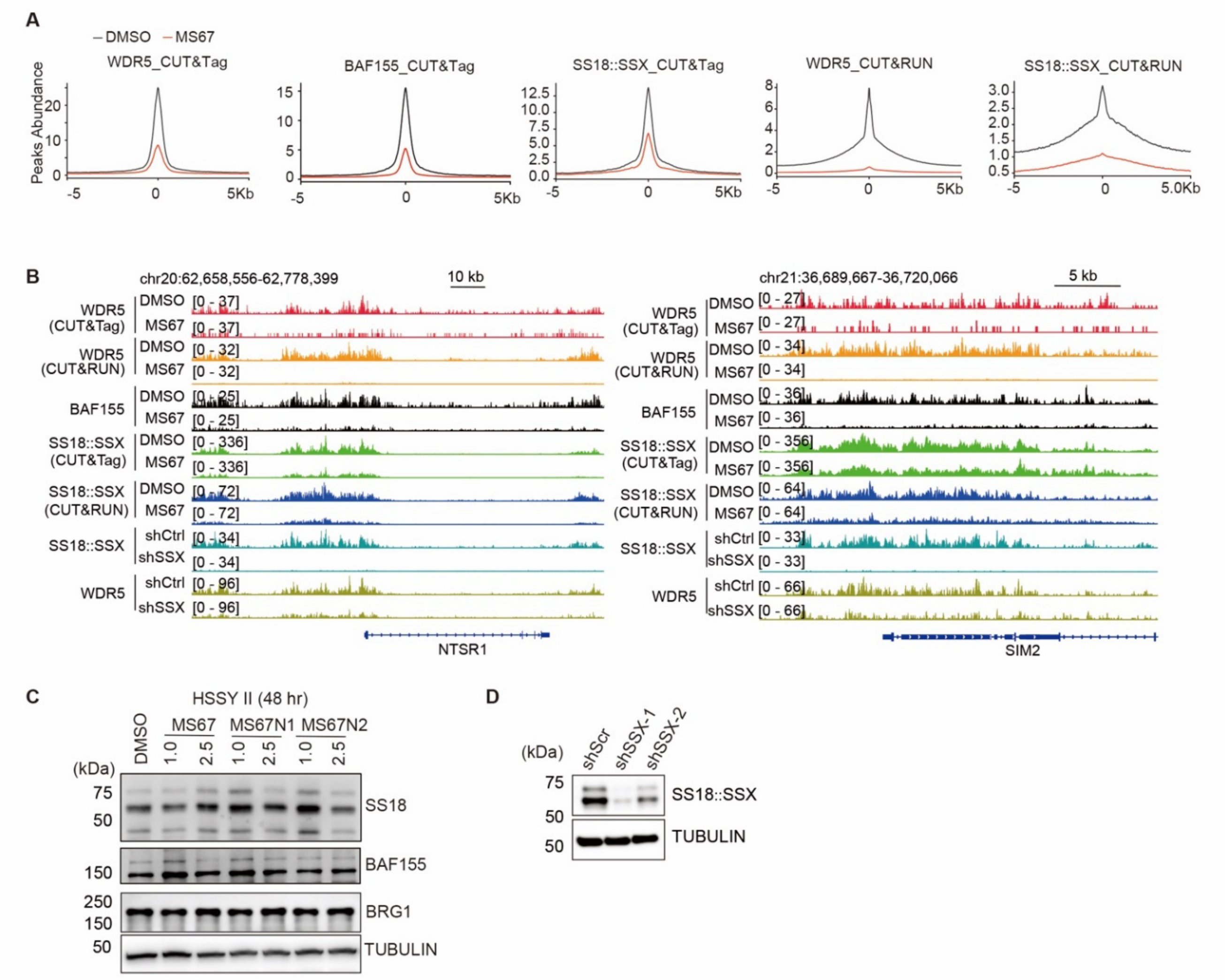

Fig. S3. WDR5-targeting PROTAC suppresses the chromatin binding of SS18::SSX and the associated SWI/SNF complexes in SS cells. (**A**) Averaged intensities of the indicated WDR5, SS18::SSX or BAF155 CUT&Tag and/or CUT&RUN signals in HSSY II cells, treated with 2.5µM of DMSO (black) or MS67 (red) for 4 days. The y-axis represents average signals across a region ±5Kb from the peak center (x-axis). (**B**) IGV views of the indicated CUT&Tag or CUT&RUN signals at *NTSR1* and *SIM2* in HSSY II cells, treated with either DMSO or MS67 (the top 10 panels), or with stable transduction of a control shRNA (shCtrl) or a SS18::SSX-targeting shRNA (shSSX; the bottom 4 panels). (**C**) Immunoblotting of SS18::SSX, SMARCC1/BAF155, SMARCA4/BRG1 and TUBULIN in HSSY II cells, treated with the indicated concentration of DMSO, MS67, MS67N1 or MS67N2 for 48 hours. (**D**) Immunoblotting of the indicated protein in HSSY II cells stably transduced with a shRNA control (shCtrl) or two different shRNAs targeting SS18::SSX (shSSX-1 or shSSX-2).

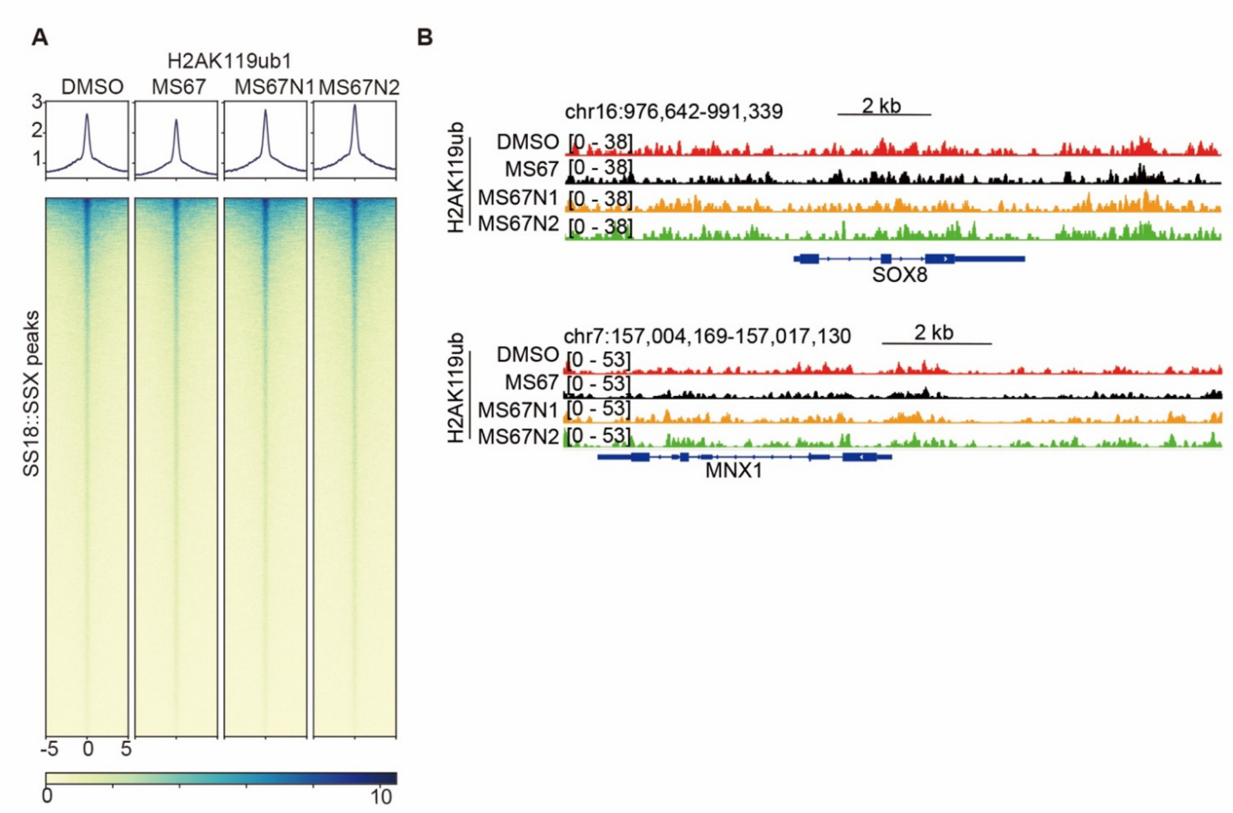

Fig. S4. WDR5 degradation did not alter the overall patterns of H2AK119ub at the SS18::SSX-targeted genomic sites. (**A** and **B**) Heatmaps showing the overall intensities of H2AK119ub signals, ± 5 kb from the centers of the called SS18::SSX peaks (**A**) and the IGV views of the indicated genes (**B**) in HSSY II cells, treated with 2.5μM of either DMSO, MS67, MS67N1 or MS67N2 for 4 days.

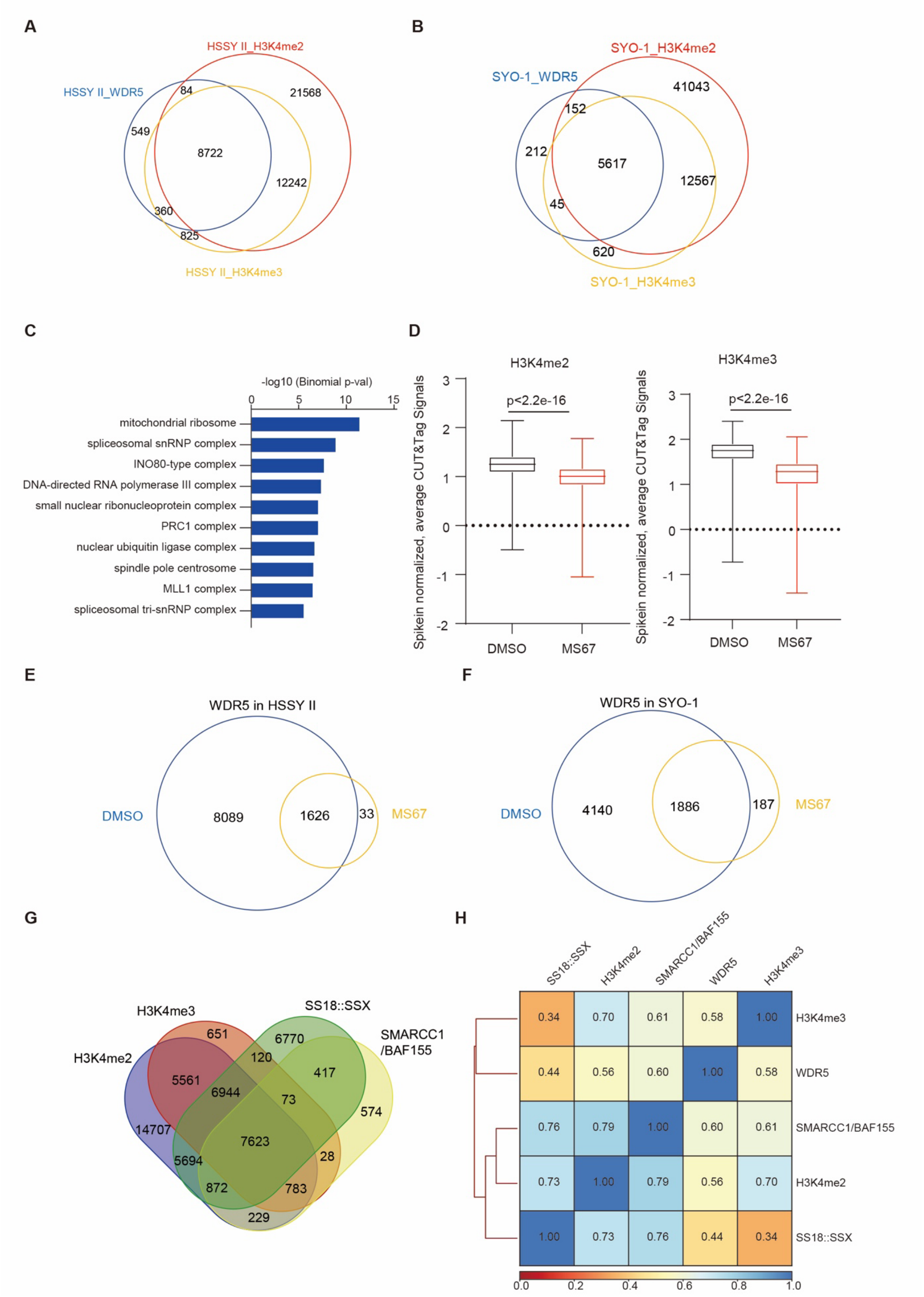

A
HSSY II_H3K4me2
HSSY II_WDR5
84
21568
549
8722
12242
360
825
HSSY II_H3K4me3
B
SYO-1_H3K4me2
SYO-1_WDR5
41043
152
212
5617
12567
45
620
SYO-1_H3K4me3
C
-log10 (Binomial p-val)
0
5
10
15
mitochondrial ribosome
spliceosomal snRNP complex
INO80-type complex
DNA-directed RNA polymerase III complex
small nuclear ribonucleoprotein complex
PRC1 complex
nuclear ubiquitin ligase complex
spindle pole centrosome
MLL1 complex
spliceosomal tri-snRNP complex
D
H3K4me2
H3K4me3
Spikein normalized, average CUT&Tag Signals
p<2.2e-16
p<2.2e-16
DMSO
MS67
DMSO
MS67
E
WDR5 in HSSY II
DMSO
8089
1626
33
MS67
F
WDR5 in SYO-1
DMSO
4140
1886
187
MS67
G
H3K4me3
SS18::SSX
SMARCC1
/BAF155
H3K4me2
651
6770
120
5561
417
6944
73
574
14707
7623
5694
28
872
783
229
H
SS18::SSX
H3K4me2
SMARCC1/BAF155
WDR5
H3K4me3
0.34 0.70 0.61 0.58 1.00 H3K4me3
0.44 0.56 0.60 1.00 0.58 WDR5
0.76 0.79 1.00 0.60 0.61 SMARCC1/BAF155
0.73 1.00 0.79 0.56 0.70 H3K4me2
1.00 0.73 0.76 0.44 0.34 SS18::SSX
0.0 0.2 0.4 0.6 0.8 1.0

Fig. S5. WDR5 PROTAC suppresses H3K4me2/3 on the WDR5 target sites in SS cells. (**A** and **B**) Venn diagram using the called WDR5, H3K4me2 and H3K4me3 peaks in either HSSY II (**A**, mock-treated) or SYO-1 cells (**B**, mock-treated). (**C**) GO analysis of the WDR5- and H3K4me3 co-bound peaks in HSSY II cells. X axis shows the -log 10 value of Binomial *P* values. (**D**) Box plots showed averaged CUT&Tag signal intensities of H3K4me2 (**D**, left panel) or H3K4me3 (**D**, right panel) in the DMSO- versus MS67-treated HSSY II cells. Wilcox test was used to generate *P* value. (**E** and **F**) Venn diagram using WDR5 peaks identified in DMSO- and MS67-treated HSSY II (**E**) or SYO-1 cells (**F**). (**G**) Venn diagram showing the overlap among the SS18::SSX, BAF155, H3K4me2 and H3K4me3 peaks in HSSY II cells (mock-treated). (**H**) Heatmap showing the Pearson correlation coefficient using the indicated CUT&Tag data in HSSY II cells (mock-treated).

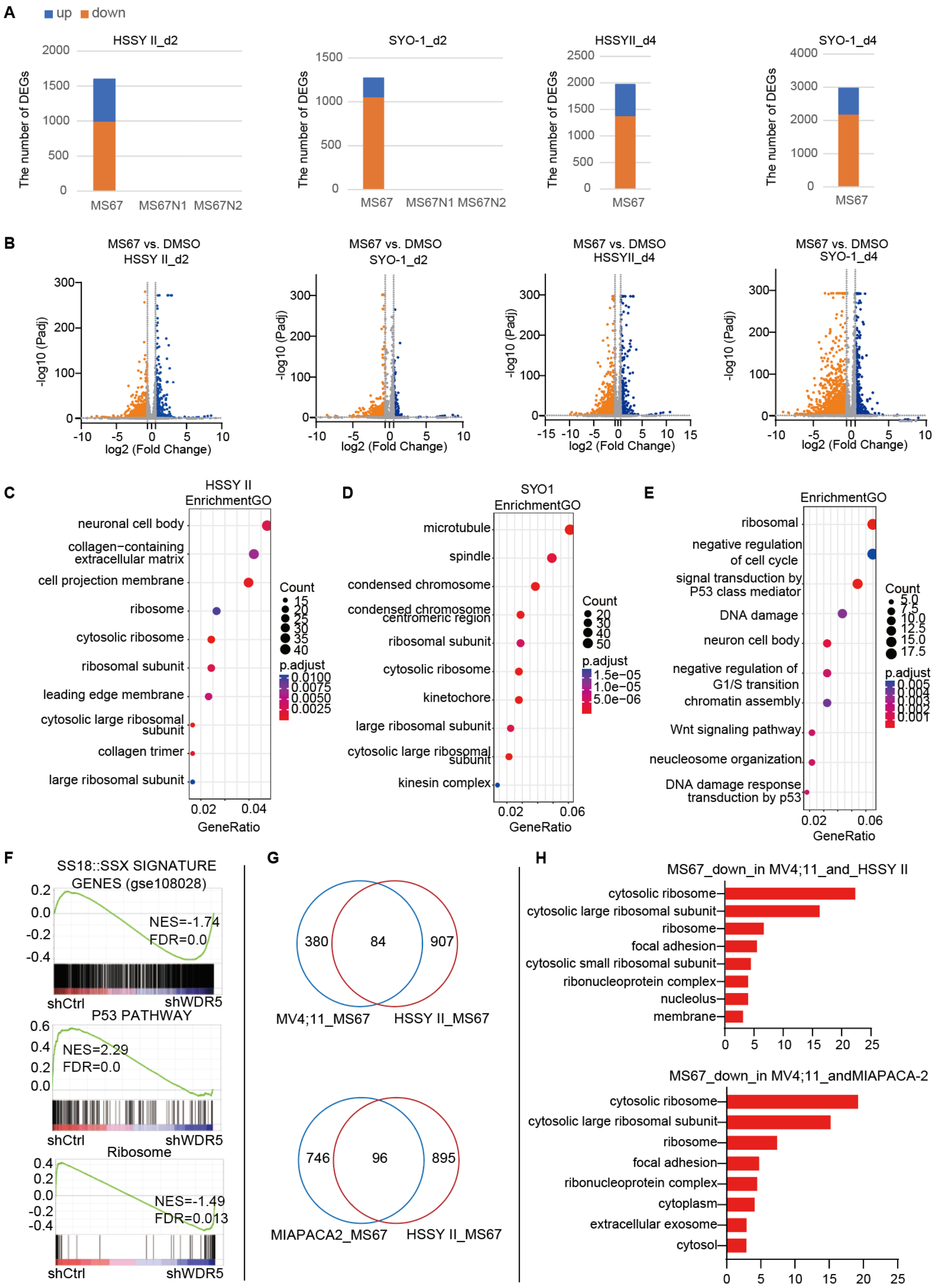

A
up down
HSSY II_d2
SYO-1_d2
HSSYII_d4
SYO-1_d4
The number of DEGs
MS67 MS67N1 MS67N2
B
MS67 vs. DMSO HSSY II_d2
MS67 vs. DMSO SYO-1_d2
MS67 vs. DMSO HSSYII_d4
MS67 vs. DMSO SYO-1_d4
-log10 (Padj)
log2 (Fold Change)
C
HSSY II EnrichmentGO
neuronal cell body
collagen−containing extracellular matrix
cell projection membrane
ribosome
cytosolic ribosome
ribosomal subunit
leading edge membrane
cytosolic large ribosomal subunit
collagen trimer
large ribosomal subunit
Count
p.adjust
GeneRatio
D
SYO1 EnrichmentGO
microtubule
spindle
condensed chromosome
condensed chromosome centromeric region
ribosomal subunit
cytosolic ribosome
kinetochore
large ribosomal subunit
cytosolic large ribosomal subunit
kinesin complex
E
EnrichmentGO
ribosomal
negative regulation of cell cycle
signal transduction by P53 class mediator
DNA damage
neuron cell body
negative regulation of G1/S transition
chromatin assembly
Wnt signaling pathway
neucleosome organization
DNA damage response transduction by p53
F
SS18::SSX SIGNATURE GENES (gse108028)
NES=-1.74
FDR=0.0
shCtrl shWDR5
P53 PATHWAY
NES=2.29
FDR=0.0
Ribosome
NES=-1.49
FDR=0.013
G
380 84 907
MV4;11_MS67 HSSY II_MS67
746 96 895
MIAPACA2_MS67 HSSY II_MS67
H
MS67_down_in MV4;11_and_HSSY II
cytosolic ribosome
cytosolic large ribosomal subunit
ribosome
focal adhesion
cytosolic small ribosomal subunit
ribonucleoprotein complex
nucleolus
membrane
MS67_down_in MV4;11_andMIAPACA-2
cytosolic ribosome
cytosolic large ribosomal subunit
ribosome
focal adhesion
ribonucleoprotein complex
cytoplasm
extracellular exosome
cytosol

Fig. S6. WDR5 PROTAC inhibits transcription of the SS18::SSX-targeted oncogenes and ribosomal protein (RP)-coding genes, leading to P53 activation. (**A**) The total number of differentially expressed genes (DEGs) identified by RNA-seq (after the spike-in control normalization) in HSSY II and SYO-1 cells, treated with 2.5 μM of the compound (either MS67, MS67N1 or MS67N2) versus DMSO for 2 (d2) or 4 days (d4). Down and up refer to down- or up-regulated transcripts after the compound versus DMSO treatment, with a DEG cutoff set to the absolute value of Log2FC over 0.58 and the p-adj value less than 0.05. (**B**) Volcano plots showing DEGs, with the down- and up-regulated ones highlighted in orange and blue color, respectively, as identified by RNA-seq (using a cutoff of the absolute value of Log2FC over 0.58 and p-adj value less than 0.05) in HSSY II and SYO-1 cells following the treatment with 2.5 μM of MS67 versus DMSO for 2 or 4 days. (**C and D**) GO analysis using the DEGs down-regulated after the MS67 versus DMSO treatment for 2 days in HSSY II (**C**) or SYO-1 cells (**D**). (**E**) GO analysis using the signature genes co-bound by WDR5, SS18::SSX and SMARCC1/BAF155 whose expression also requires WDR5 in HSSY II cells, as defined with the WDR5 PROTAC treatment versus mock in **Fig. 5K**. (**F**) GSEA reveals the indicated pathway enrichment using the RNA-seq profiles of HSSY II cells stably transduced with a control shRNA (shCtrl) versus a shRNA targeting WDR5 (shWDR5). (**G**) Venn diagram using DEGs identified by RNA-seq in HSSY II, MV4;11 or MIA PaCa-2 cells to be downregulated after the treatment with MS67 versus DMSO. RNA-seq profiles of MV4;11 and MIA PaCa-2 cells are based on a previous work (28). DEG is defined with a cutoff of the absolute value of Log2FC over 0.58 and p-adj value less than 0.05. (**H**) GO analysis using the MS67-downregulated DEGs common to HSSY II and MV4;11 cells (top panel), or to HSSY II and MIA PaCa-2 cells (bottom panel).

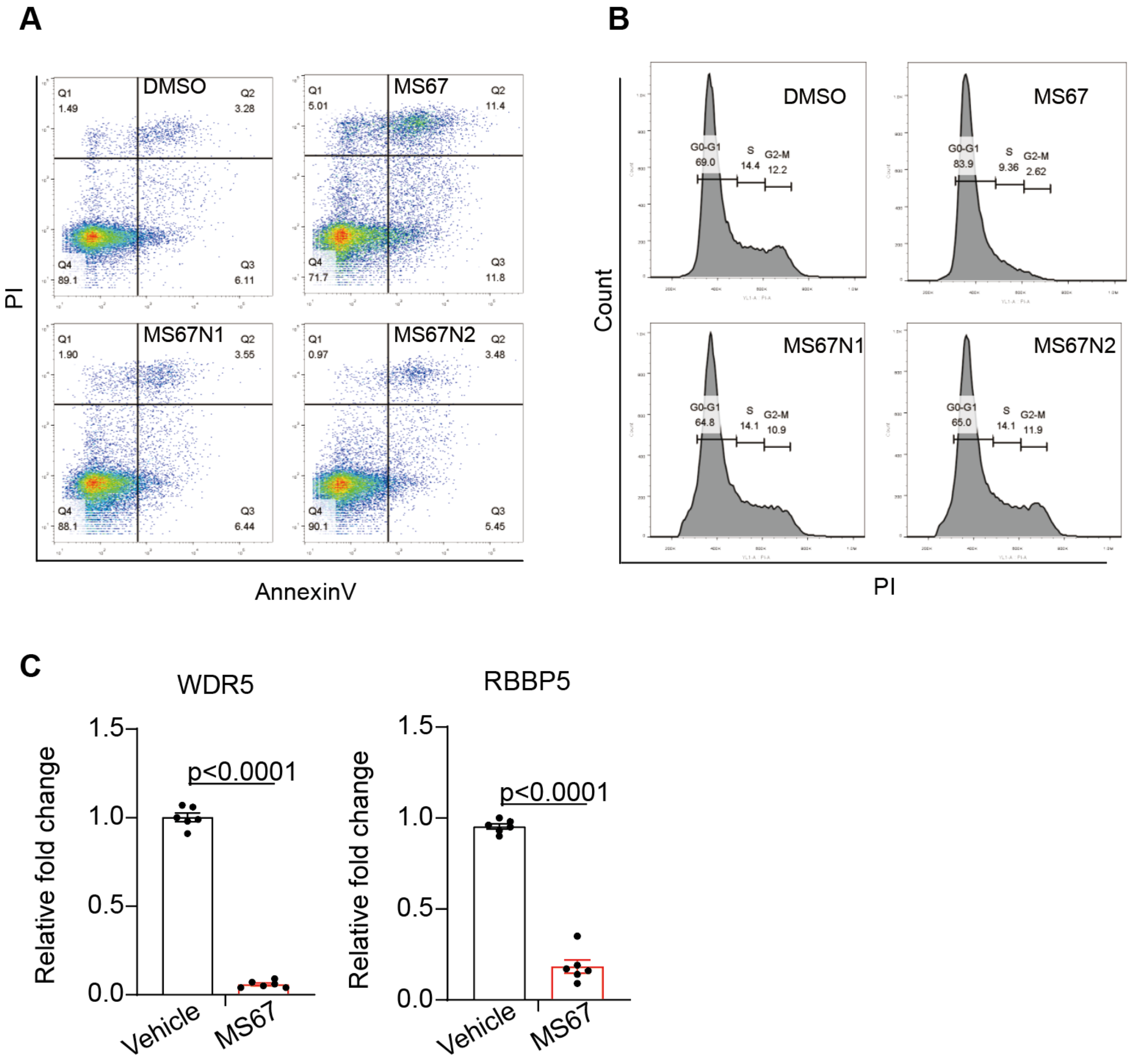

Fig. S7. WDR5 PROTAC induces SS cell senescence and apoptosis *in vitro* and suppresses SS growth *in vivo*. (**A and B**) Representative FACS profiles (**A**) showing the Annexin V and propidium iodide (PI) staining on x-axis and y-axis, respectively, to score the apoptotic cell abundance, as well as the PI-based FACS to score the cells in different cell cycle stages (**B**), in HSSY II cells after the treatment with 2.5 μM of the indicated compound for 48 hrs. (**C**) Plots of the WDR5 and RBBP5 protein levels in the CDX samples isolated 2 hours after the last dose from the vehicle- (n = 6) or MS67-treated (n = 6) NSG mice. Quantification is based on the WB results shown in **Fig. 7E**. For all relevant figures, data are represented as mean ± SEM. *P* values were calculated with two tail Student t-test.

Table S1. RNA-seq defines the differentially expressed genes (DEGs) in HSSY II cells after the treatment with 2.5 uM of the compound (MS67, MS67N1 or MS67N2) versus DMSO for 2 or 4 days, either up- or down-regulated. The cut-off of DEGs is set at the absolute Log2 value of fold-change greater than 0.58 and the p-adj value less than 0.05.

Table S2. RNA-seq defines the DEGs in SYO-I cells after the treatment with 2.5 uM of the compound (MS67, MS67N1 or MS67N2) versus DMSO for 2 or 4 days, either up- or down-regulated. The cut-off of DEGs is set at the absolute Log2 value of fold-change greater than 0.58 and the p-adj value less than 0.05.

Table S3. RNA-seq defines the DEGs in HSSY II cells after the shRNA-mediated WDR5 knockdown (KD) (shWDR5-1 or shWDR5-2) versus control shRNA (shCtl), either up- or down-regulated. The cut-off of DEGs is set at the absolute Log2 value of fold-change greater than 0.58 and the p-adj value less than 0.05.

Table S4. Sequence information of primers used for plasmid construction or RT-qPCR.

<u>**Plasmid construction**</u>	
pLKO.1-shWDR5-1-F	CCGGGCCTCCTCTCTGAAGATGATTCTCGAGAATCATCTTCAGAGAGGAGGCTTTTTG
pLKO.1-shWDR5-1-R	AATTCAAAAAGCCTCCTCTCTGAAGATGATTCTCGAGAATCATCTTCAGAGAGGAGGC
pLKO.1-shWDR5-2-F	CCGGGCTCAGAGGATAACCTTGTTTCTCGAGAAACAAGGTTATCCTCTGAGCTTTTTG
pLKO.1-shWDR5-2-R	AATTCAAAAAGCTCAGAGGATAACCTTGTTTCTCGAGAAACAAGGTTATCCTCTGAGC
pLKO.1-shSSX-1-F	CCGGAGAAAGCAGCTGGTGATTTATCTCGAGATAAATCACCAGCTGCTTTCTTTTTTG
pLKO.1-shSSX-1-R	AATTCAAAAAAGAAAGCAGCTGGTGATTTATCTCGAGATAAATCACCAGCTGCTTTCT
pLKO.1-shSSX-2-F	CCGGCAGTCACTGACAGTTAATAAACTCGAGTTTATTAACTGTCAGTGACTGTTTTTG
pLKO.1-shSSX-2-R	AATTCAAAAACAGTCACTGACAGTTAATAAACTCGAGTTTATTAACTGTCAGTGACTG
pLKO.1-shScr-F	CCGGCCTAAGGTTAAGTCGCCCTCGCTCGAGCGAGGGCGACTTAACCTTAGGTTTTTG
pLKO.1-shScr-R	AATTCAAAAACCTAAGGTTAAGTCGCCCTCGCTCGAGCGAGGGCGACTTAACCTTAGG
<u>**RT-qPCR**</u>	
h-SNHG15-f	GCTGAGGTGACGGTCTCAAA
h-SNHG15-r	GCCTCCCAGTTTCATGGACA
h-GAPDH-f	GAAGGTGAAGGTCGGAGTC
h-GAPDH-r	GAAGATGGTGATGGGATTTC
h-RPL27-f	TCCGGACGCAAAGCTGTCATCG
h-RPL27-r	TCTTGCCCATGGCAGCTGTCA
h-RPL7-f	AAGATCAAGCGCCTGAGAAAG
h-RPL7-r	TGCAGGTACATAGAAGTTGCCA
h-RPL35-f	AGCTCTCTAAGATCCGAGTCG

h-RPL35-r	GAACACGGGCAATGGATTTCC
h-SNHG15-f	GCTGAGGTGACGGTCTCAAA
h-SNHG15-r	GCCTCCCAGTTTCATGGACA
h-RPS10-f	ATGTTGATGCCTAAGAAGAACCG
h-RPS10-r	CGTAGCCTCGGGACTTGAGA
h-LHX3-f	CAGTATTTCCGCAACATGAAGC
h-LHX3-r	GCTCCCGTAGAGGCCATTG
h-SIM2-f	CCATTTAGGCTTATCCCAGGTG
h-SIM2-r	GGTCATCTCATCGTGGTCAGA
h-MNX1-f	CTCCTACTCGTACCCGCAG
h-MNX1-r	TTGAAGTCGGGCATCTTAGGC
h-NTSR1-f	ACCGTCAAGGTCGTCATACAG
h-NTSR1-r	TGATGGTGTTCAGGACCGAGA
h-WNT7B-f	GAAGCAGGGCTACTACAACCA
h-WNT7B-r	CGGCCTCATTGTTATGCAGGT
h-FZD10-f	GCTCATGGTGCGTATCGGG
h-FZD10-r	GAGGCGTTCGTAAAAGTAGCA
h-FGF3-f	GACGACTCTATGCTTCGGAGC
h-FGF3-r	AGGCGTACTAGACACCGTCC
h-SOX8-f	CAAGGGCTACGACTGGAGTCT
h-SOX8-r	CATGCGGCTTGGCTTTGAG
h-TP53-f	ACAGCTTTGAGGTGCGTGTTT
h-TP53-r	CCCTTTCTTGCGGAGATTCTCT
h-CDKN1A-f	TGTCCGTCAGAACCCATGC
h-CDKN1A-r	AAAGTCGAAGTTCCATCGCTC

Fig. 1C(left panel)

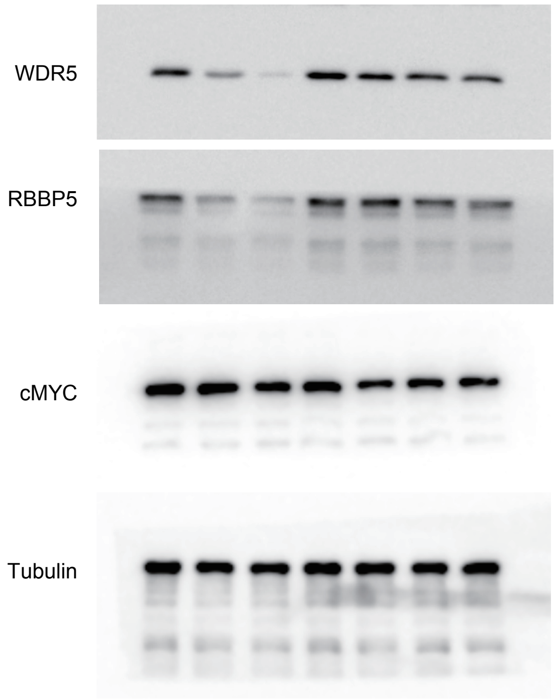

Fig. 1C(right panel)

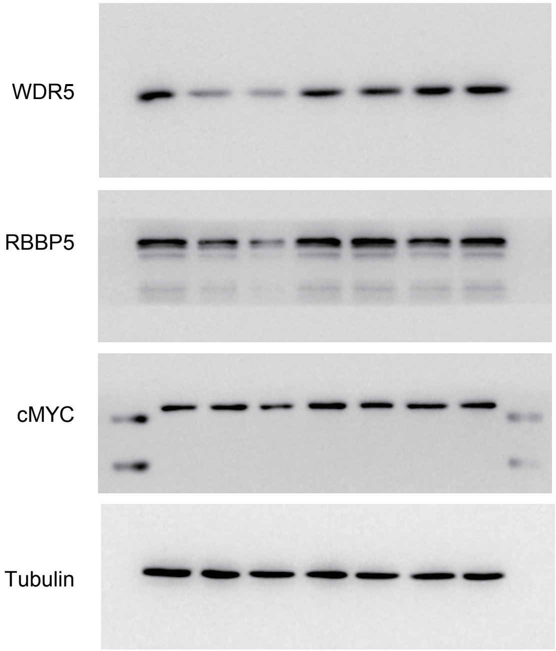

Fig. 1D (left panel)

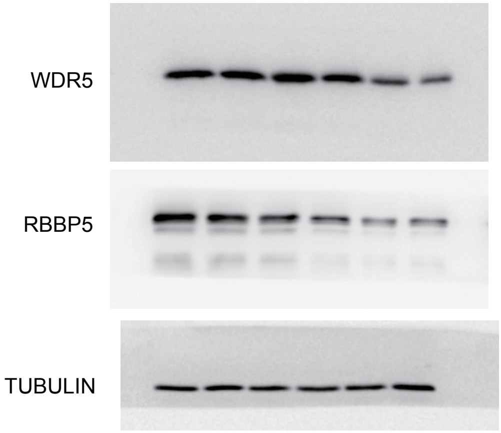

Fig. 1D (right panel)

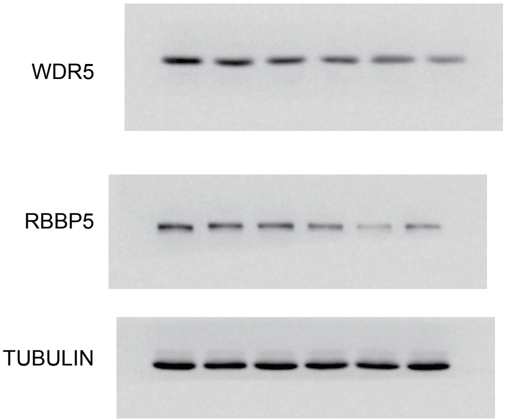

Fig. 2B

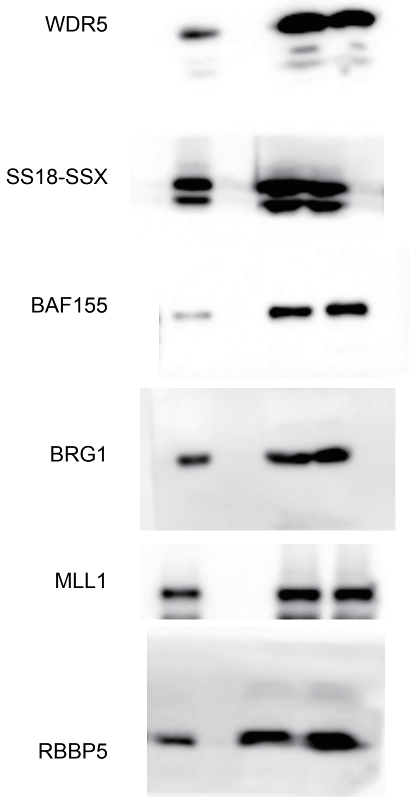

Fig. 2C

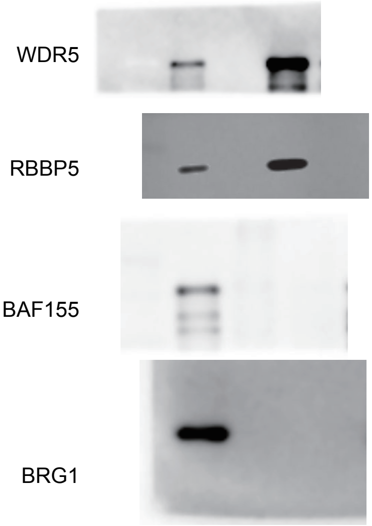

Fig. 3E

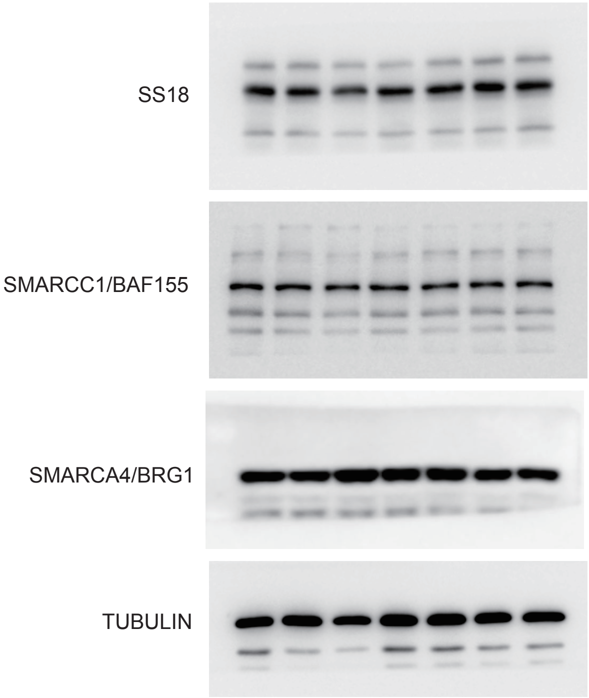

Fig. 3F

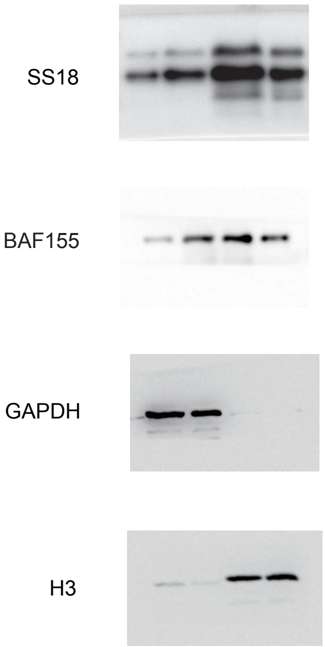

Fig. 3G

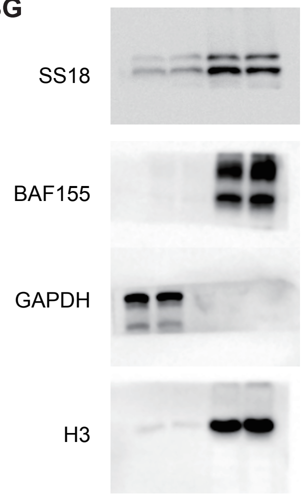

Fig. 3H

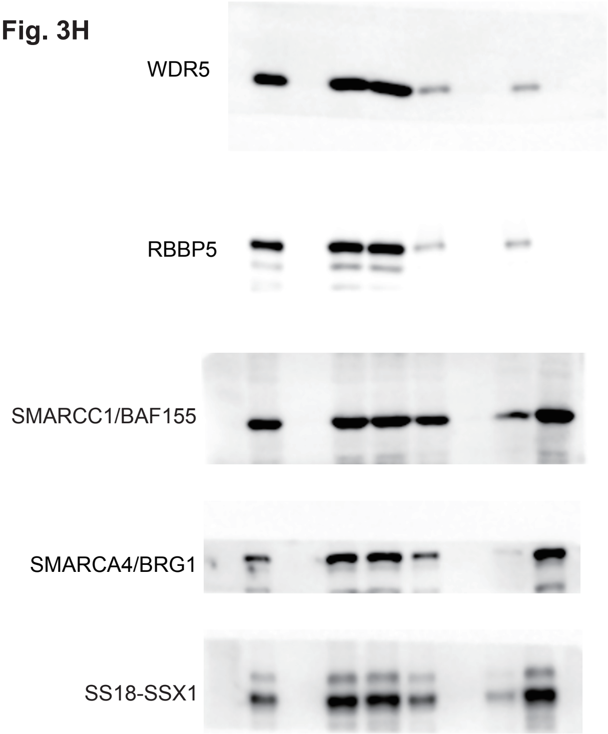

Fig. 4A

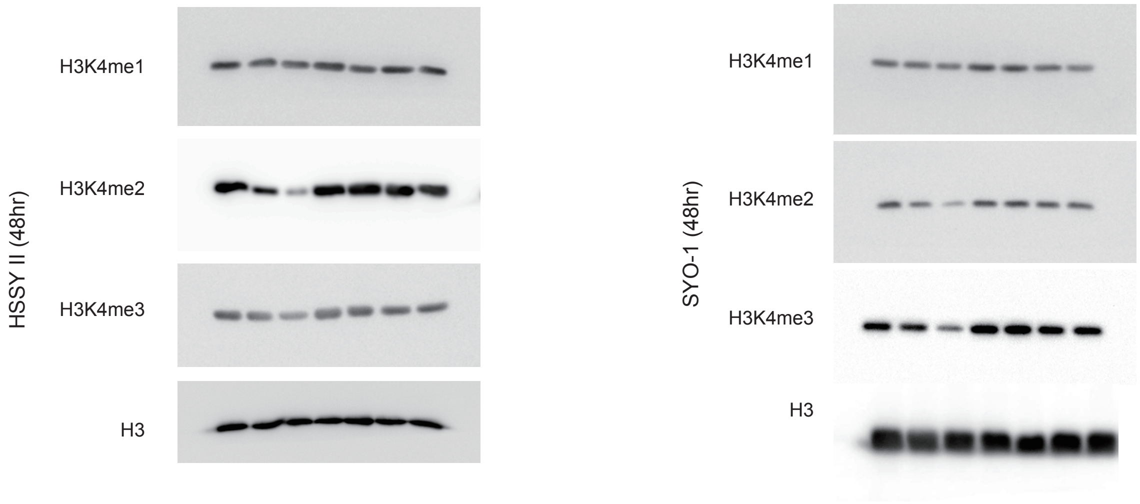

Fig. 6A

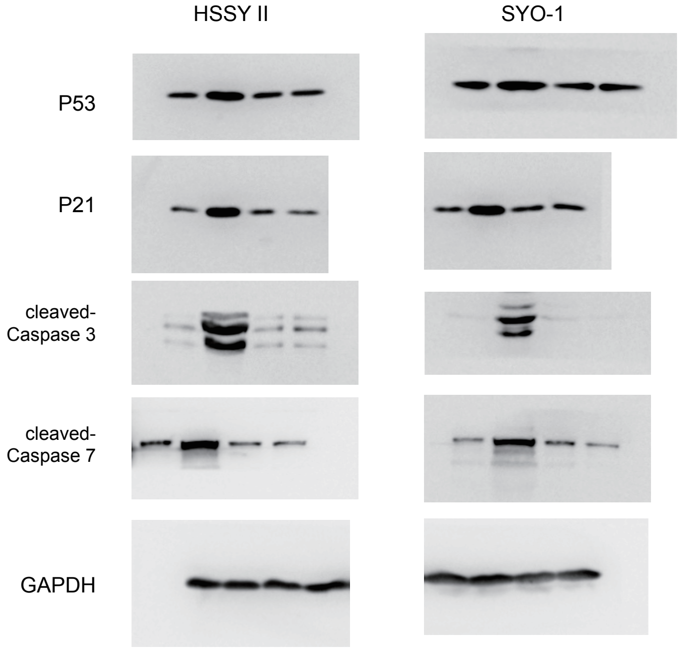

Fig. 6F

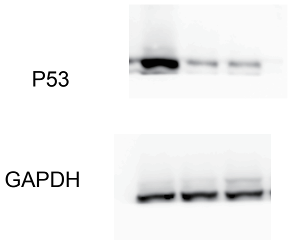

Fig. 7E

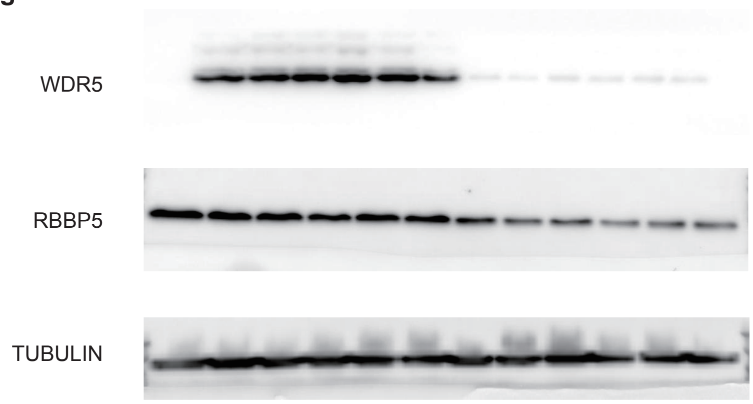

Fig. S1C

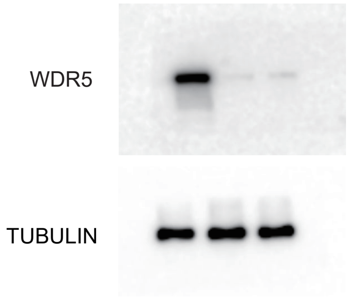

Fig. S1F

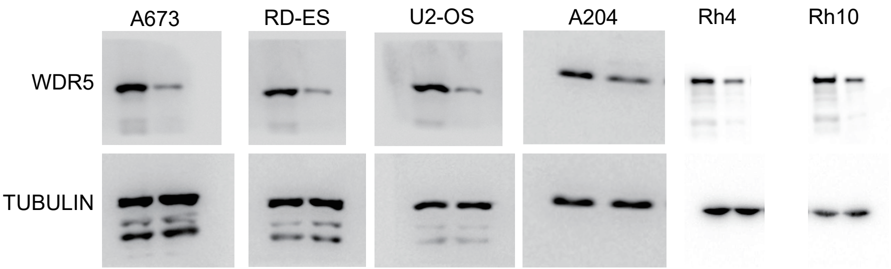

Fig. S1H

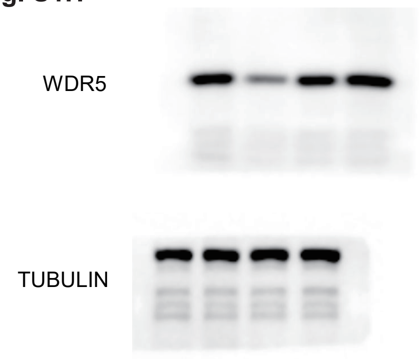

Fig. S3C

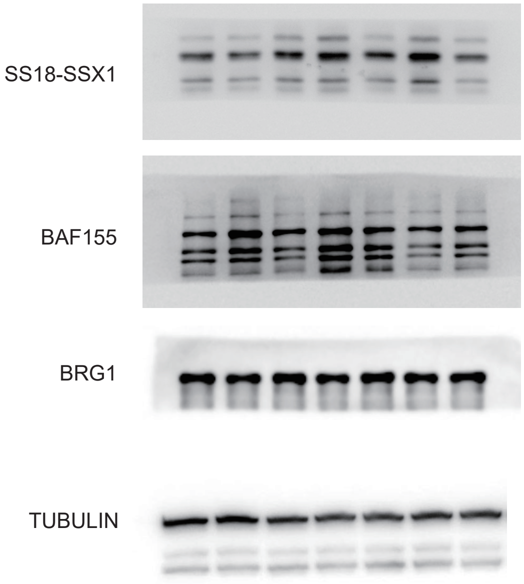

Fig. S3D

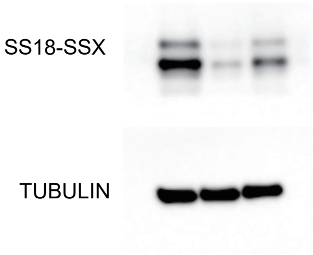

General Scheme of Synthetic route for MS67N2.

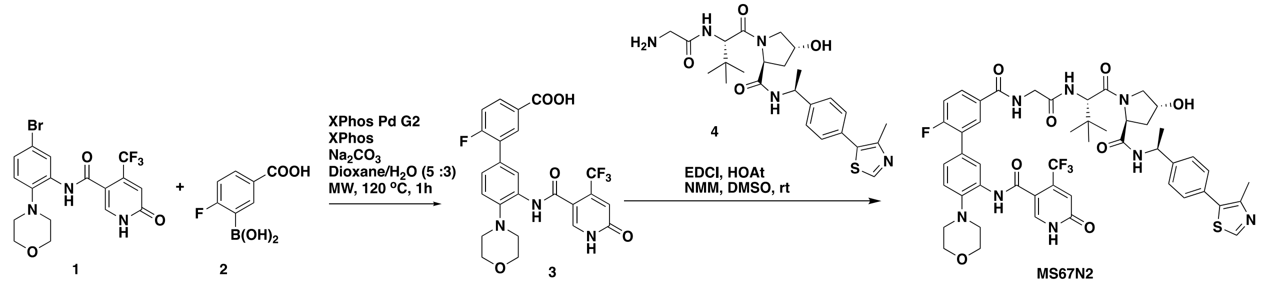

^{1}H-NMR, ^{13}C-NMR and HPLC-LCMS spectra of MS67N2

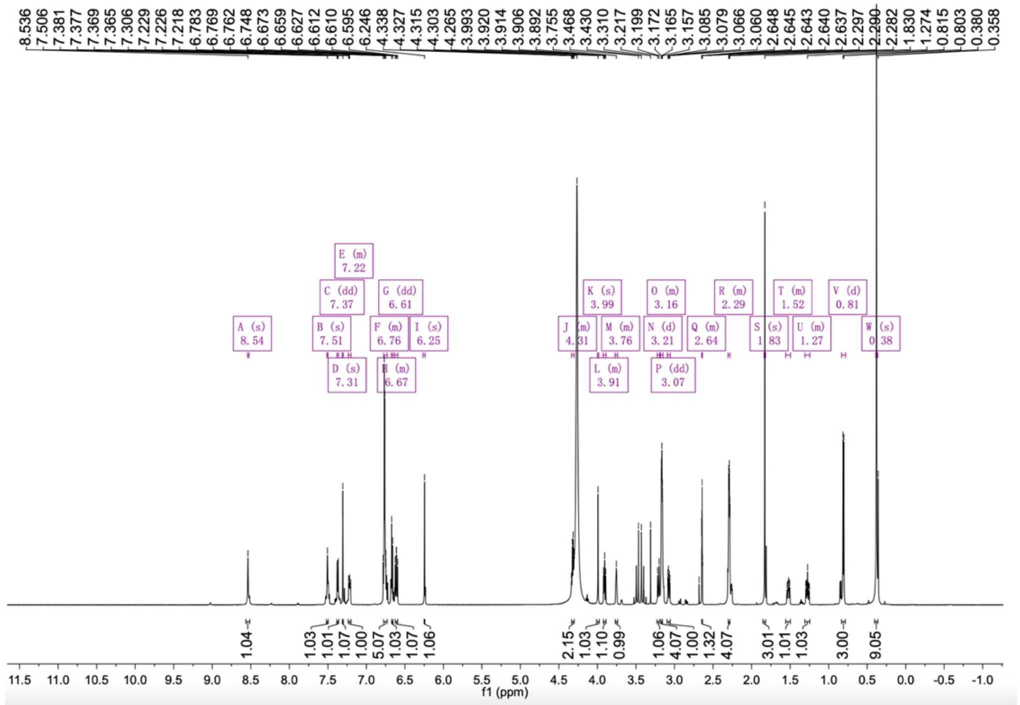

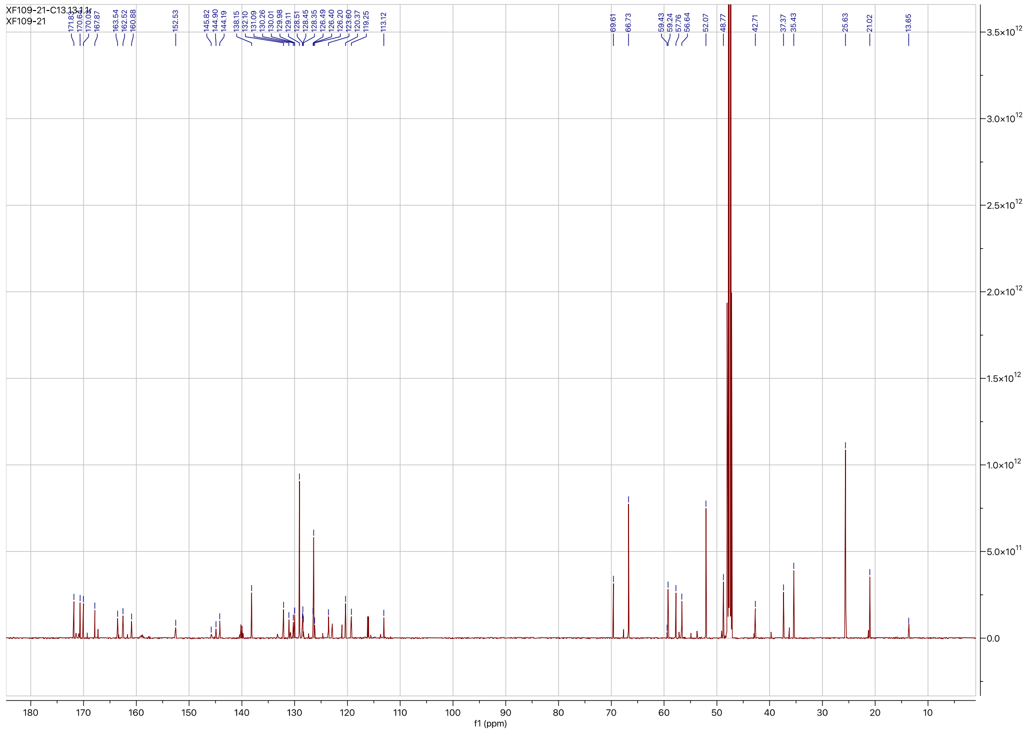

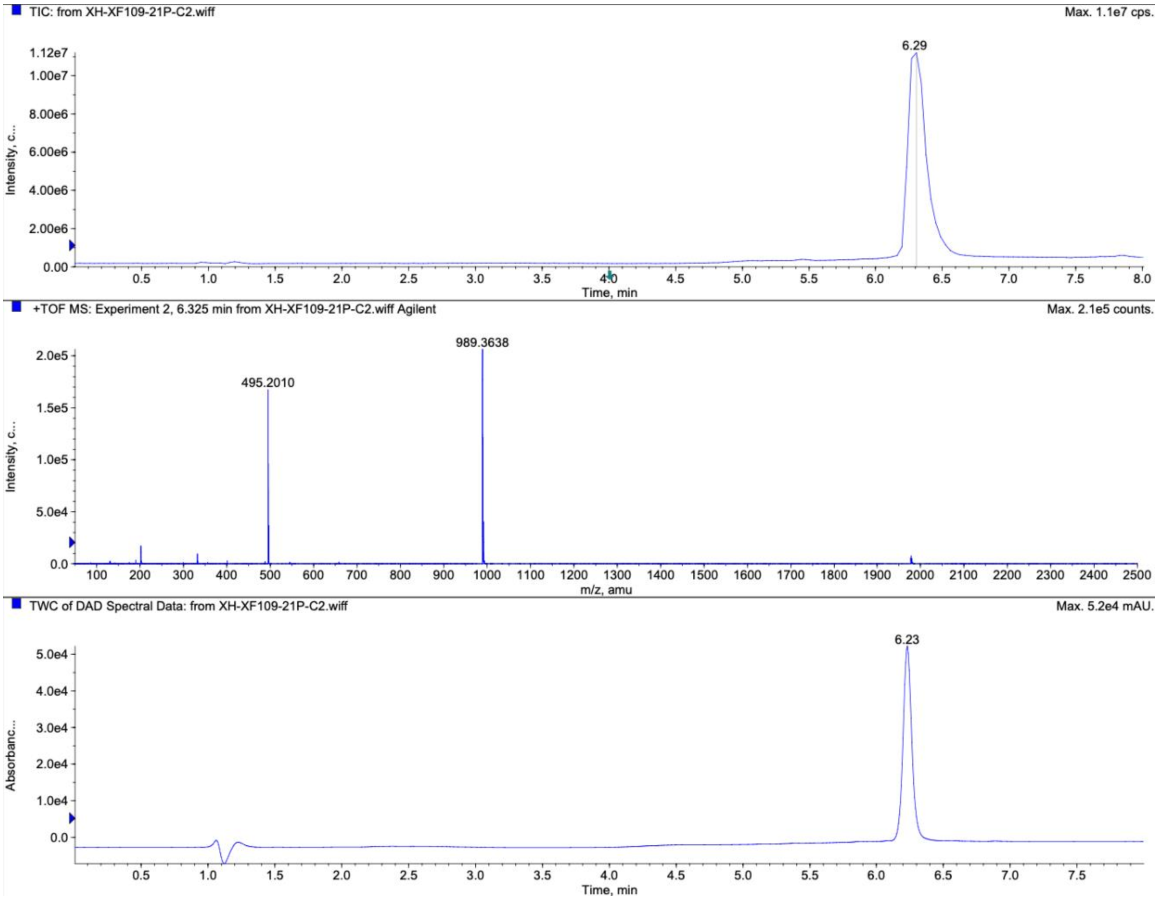

TIC: from XH-XF109-21P-C2.wiff
Max. 1.1e7 cps.
6.29
Intensity, c...
Time, min
+TOF MS: Experiment 2, 6.325 min from XH-XF109-21P-C2.wiff Agilent
Max. 2.1e5 counts.
989.3638
495.2010
Intensity, c...
m/z, amu
TWC of DAD Spectral Data: from XH-XF109-21P-C2.wiff
Max. 5.2e4 mAU.
6.23
Absorbanc...
Time, min
